# Author Correction: Freshwater genome-reduced bacteria exhibit pervasive episodes of adaptive stasis

**DOI:** 10.1038/s41467-024-49328-4

**Published:** 2024-06-06

**Authors:** Lucas Serra Moncadas, Cyrill Hofer, Paul-Adrian Bulzu, Jakob Pernthaler, Adrian-Stefan Andrei

**Affiliations:** 1 Limnological Station, Department of Plant and Microbial Biology, University of Zurich, Kilchberg, Switzerland; 2 Department of Aquatic Microbial Ecology, Institute of Hydrobiology, Biology Centre of the Czech Academy of Sciences, České Budějovice, Czech Republic

**Keywords:** Water microbiology, Metagenomics, Bacterial genomics, Bacterial evolution, Microbial ecology

Correction to: *Nature Communications* 10.1038/s41467-024-47767-7, published online 23 April 2024

In the Acknowledgements section of this article, the grant number relating to Swiss National Science Foundation given for Prof. Jakob Pernthaler was missing and should have been 10000877. The original article has been corrected.

