# Supplementary material for: Freshwater genome-reduced bacteria exhibit pervasive episodes of adaptive stasis
Source: Nat Commun. 2024 Apr 23;15:3421. doi: 10.1038/s41467-024-47767-7 (PMC11039613; doi:10.1038/s41467-024-47767-7)
Supplement: Supplementary file 1 — Supplementary Information [file 41467_2024_47767_MOESM1_ESM.pdf]

Freshwater genome-reduced bacteria exhibit pervasive episodes of adaptive stasis

Lucas Serra Moncadas[1], Cyrill Hofer[1], Paul-Adrian Bulzu[2], Jakob Pernthaler[1], Adrian-Stefan Andrei[1,*]

[1]Limnological Station, Department of Plant and Microbial Biology, University of Zurich; Kilchberg 8802, Switzerland.

[2]Department of Aquatic Microbial Ecology, Institute of Hydrobiology, Biology Centre of the Academy of Sciences of the Czech Republic; České Budějovice 37005, Czech Republic.

*Corresponding author. Email: stefan.andrei@limnol.uzh.ch

Supplementary material

Supplementary Table 1

Supplementary Figures S1-9

Supplementary Table 1.

Mock community	Sequencing Technology	Average ANI*	Average Coverage
Bacillus	Illumina	100	97.91
Enterococcus	Illumina	100	97.17
Escherichia	Illumina	99.95	85.95
Lactobacillis	Illumina	99.99	91.75
Listeria	Illumina	99.99	97.25
Pseudomonas	Illumina	99.89	96.97
Salmonella	Illumina	99.92	87.04
Staphylococcus	Illumina	99.97	95.88

*ANI: average nucleotide identity.

The accuracy of the DNA extraction, sequencing, and metagenomic assembly was evaluated by calculating the average nucleotide identity between the generated draft genomes and the provided reference genomes of the ZymoBIOMICS Microbial Community Standard D6300. The draft genomes of the eight bacteria exhibited a median average nucleotide identity of 99.98%, with a median coverage value of 96.425% compared to the reference.

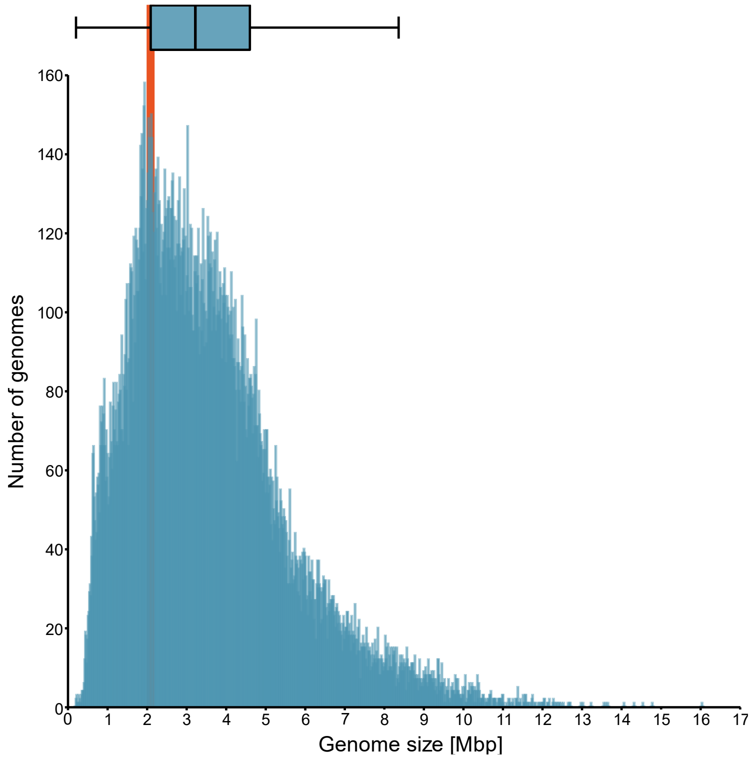

Supplementary Figure S1. Prokaryotic genome size distribution. The figure depicts the size distribution of 31,910 representative genomes present in the GTDB R05-RS95 database. The vertical red line represents the first quantile at 2.1 Mbp, which was used in this study as a threshold to define small and large genome species. The central line across the boxplot identifies the median, marking the dataset's midpoint. The box itself demarcates the interquartile range, extending from the first quartile to the third quartile, encapsulating the central 50% of the data. The whiskers project from the box to the furthest data points not categorized as outliers and show the spread of the main body of the dataset. Raw data is provided as a Source Data file.

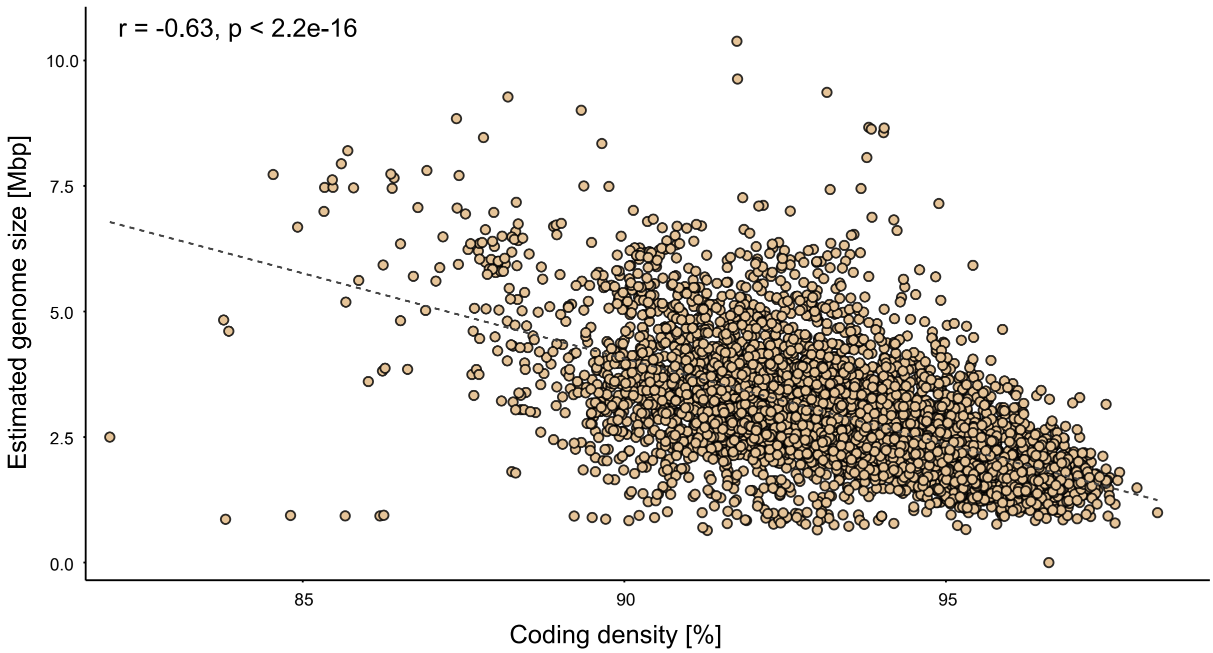

Supplementary Figure S2. Estimated genome size and coding density relationship. Linear regression between the estimated genome size and coding density in the pdCEL database (n = 5519). The whiskers project from the box to the furthest data points not categorized as outliers and show the spread of the main body of the dataset. Raw data is provided as a Source Data file.

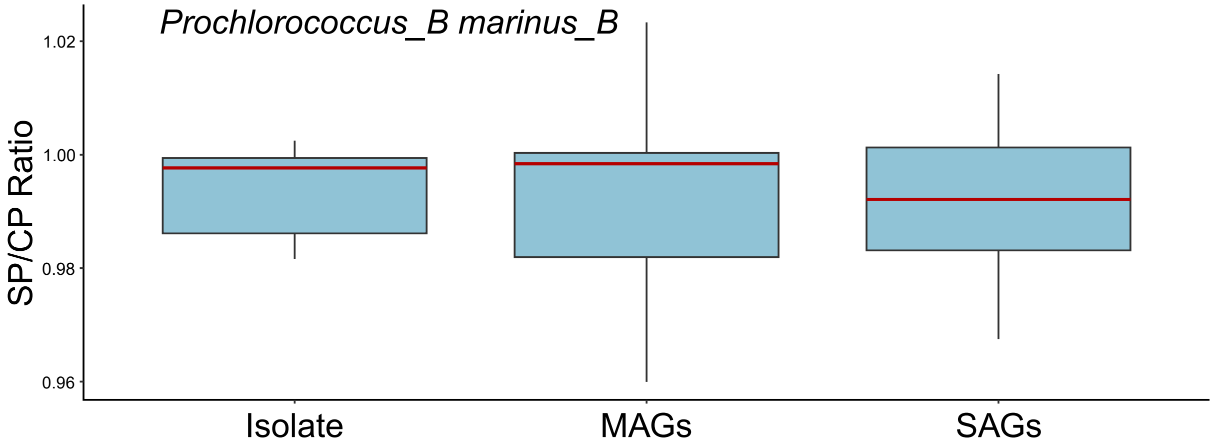

Supplementary Figure S3. SP/CP ratio benchmarking. Intraspecific index obtained by dividing median amino acid similarity values of secreted and cytoplasmic proteomes for Prochlorococcus_B marinus_B species categorized into MAGs (n = 5), SAGs (n = 10), and cultured representatives (i.e. isolates; n = 5). No statistical difference was observed between the three categories (Two-way ANOVA; df = 2; f-value = 0.3852; p-value = 0.6811). The central line across the boxplots shows the median, marking the dataset's midpoint. The box itself demarcates the interquartile range, extending from the first quartile to the third quartile, encapsulating the central 50% of the data. The whiskers project from the box to the furthest data points not categorized as outliers and show the spread of the main body of the dataset. Raw data is provided as a Source Data file.

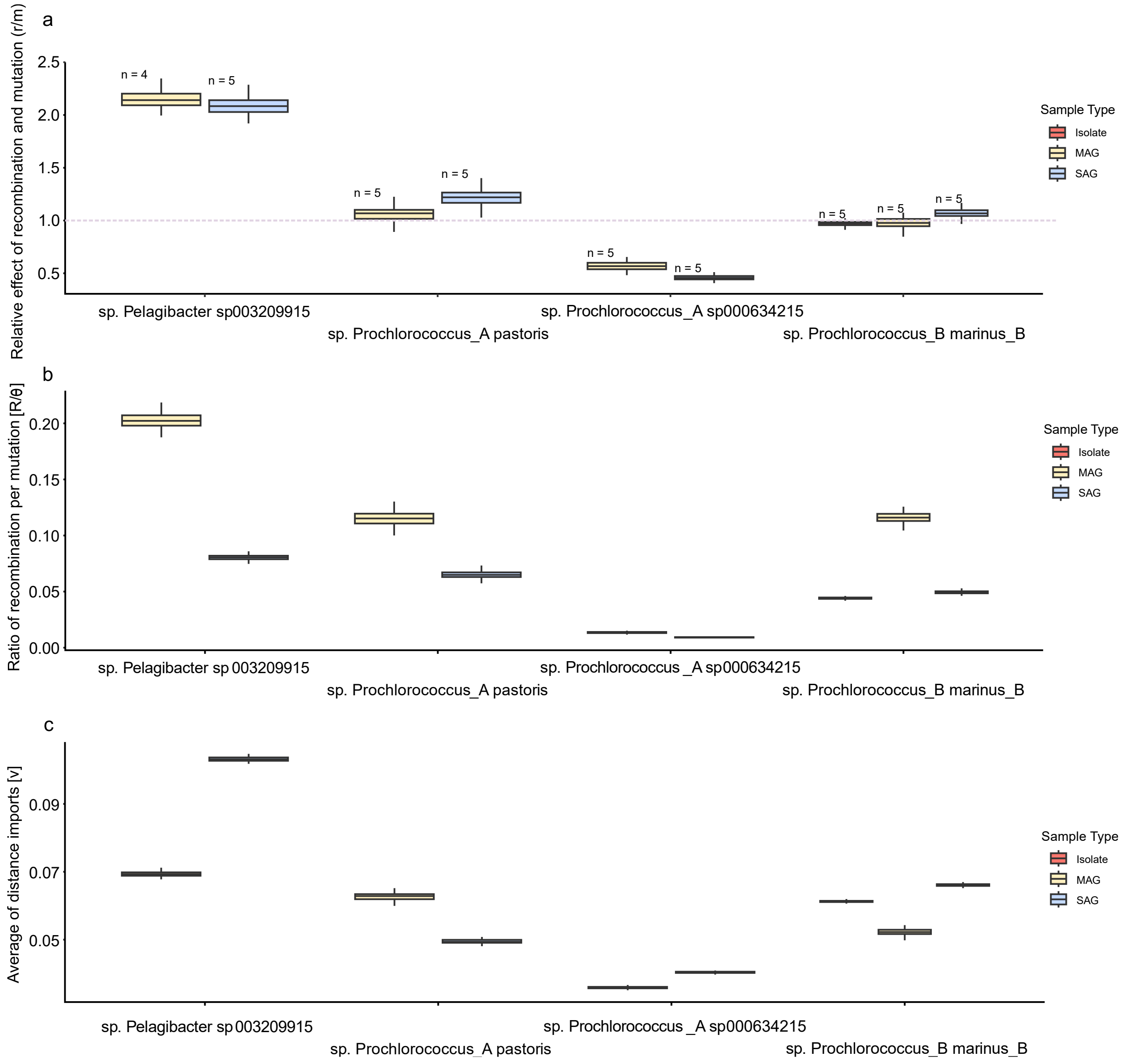

Supplementary Figure S4. Recombination and mutation rate measurements for MAGs, SAGs, and isolated representatives. a) Boxplots illustrate the relative effect of recombination and mutation for each species, categorized as MAG (light yellow), SAG (light blue), and isolates (red). The numbers above each boxplot represent the number of genomes used for species within each category. The grey line serves as an indicator representing a ratio where the effect of recombination equals mutations. b) Ratio of recombination to mutation for the above-mentioned categories. Similarly, each species is categorized into MAG, SAG, and isolate, with numbers above the boxplots representing the number of genomes used for these categories. c) Average distance of imports in the same fashion as the first two panels. The central line across the boxplots shows the median, marking the dataset's midpoint. The box itself demarcates the interquartile range, extending from the first quartile to the third quartile, encapsulating the central 50% of the data. The whiskers project from the box to the furthest data points not categorized as outliers and show the spread of the main body of the dataset. Raw data is provided as a Source Data file.

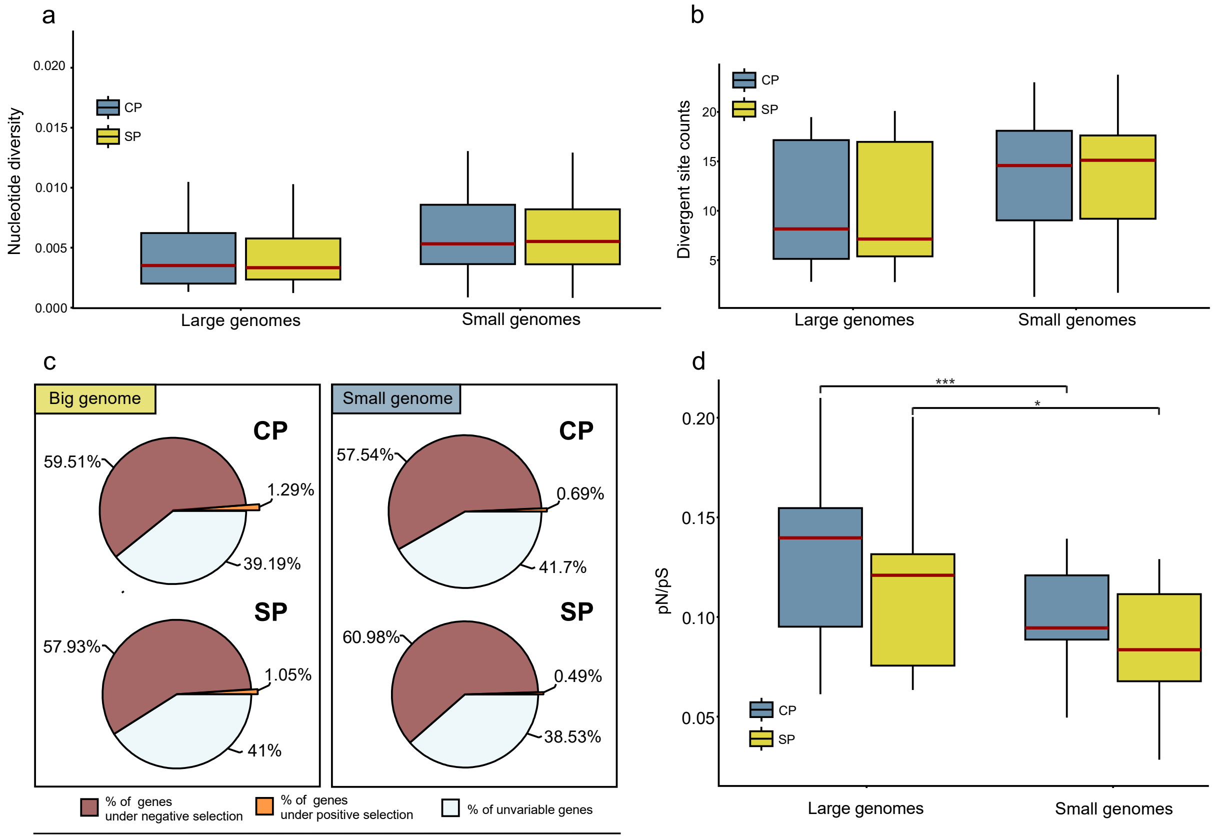

Supplementary Figure S5. InStrain results. a) Nucleotide diversity for both large and small genome species. Two boxplots are presented for each category, distinguishing the secreted proteome (SP) in yellow and the cytoplasmatic proteome (CP) in blue. b) Divergent site counts in the same fashion as panel a). c) Percentages of genes under negative (red) and positive (orange) selection within the SPs and CPs. d) pN/pS ratios for each species category in the same fashion as panel a) (Large CP n = 25903, SP n = 4436; Small CP n = 10132, SP n = 815). Statistical differences are represented through stars (Pairwise wilcox test pN/pS SP p-value = 0.00101; pN/pS CP p-value < 2.2e-16) The central line across the boxplots shows the median, marking the dataset's midpoint. The box itself demarcates the interquartile range, extending from the first quartile to the third quartile, encapsulating the central 50% of the data. The whiskers project from the box to the furthest data points not categorized as outliers and show the spread of the main body of the dataset. Raw data is provided as a Source Data file.

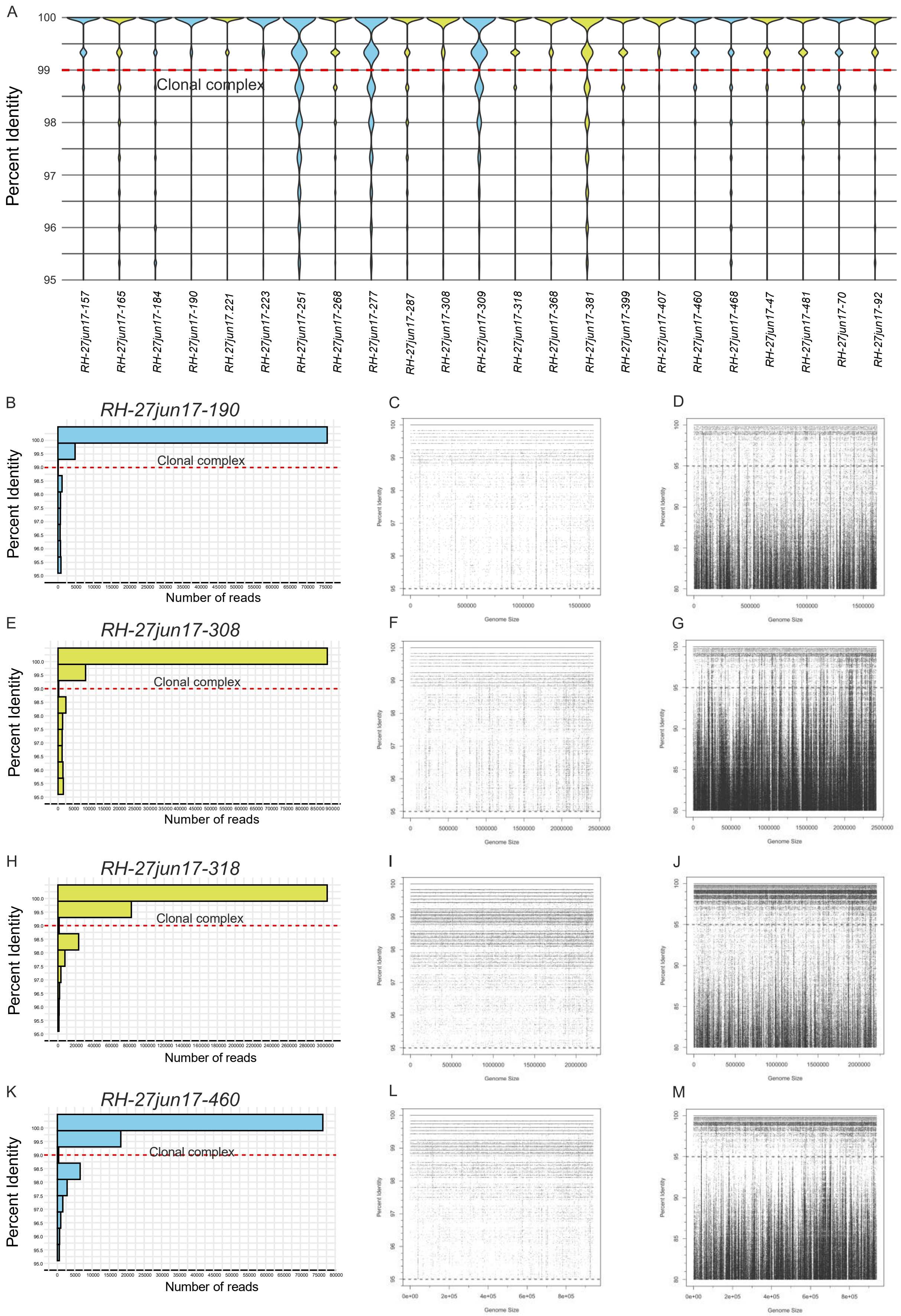

Supplementary Figure S6. Metagenomic recruitment plots. Panel A showcases the distribution of quality-filtered Illumina shotgun reads against metagenome-assembled

genomes (MAGs) derived from the same metagenomic dataset. The horizontal red line delineates clonal complexes within intraspecies boundaries (95-100% sequence identity). The yellow and blue colours distinguish between large and small MAGs, respectively. Panels B through M delve into the dynamics of co-occurring microbial populations at the time of sampling, offering a granular view of the community structure. Panels B, E, H, and K focus specifically on the quantity of mapped reads correlating to distinct identity percentages, offering insights into the genetic diversity and sequence similarity within the sampled populations. Panels C, D, F, G, I, J, L, and M map these reads' locational positions across the various analysed MAGs.

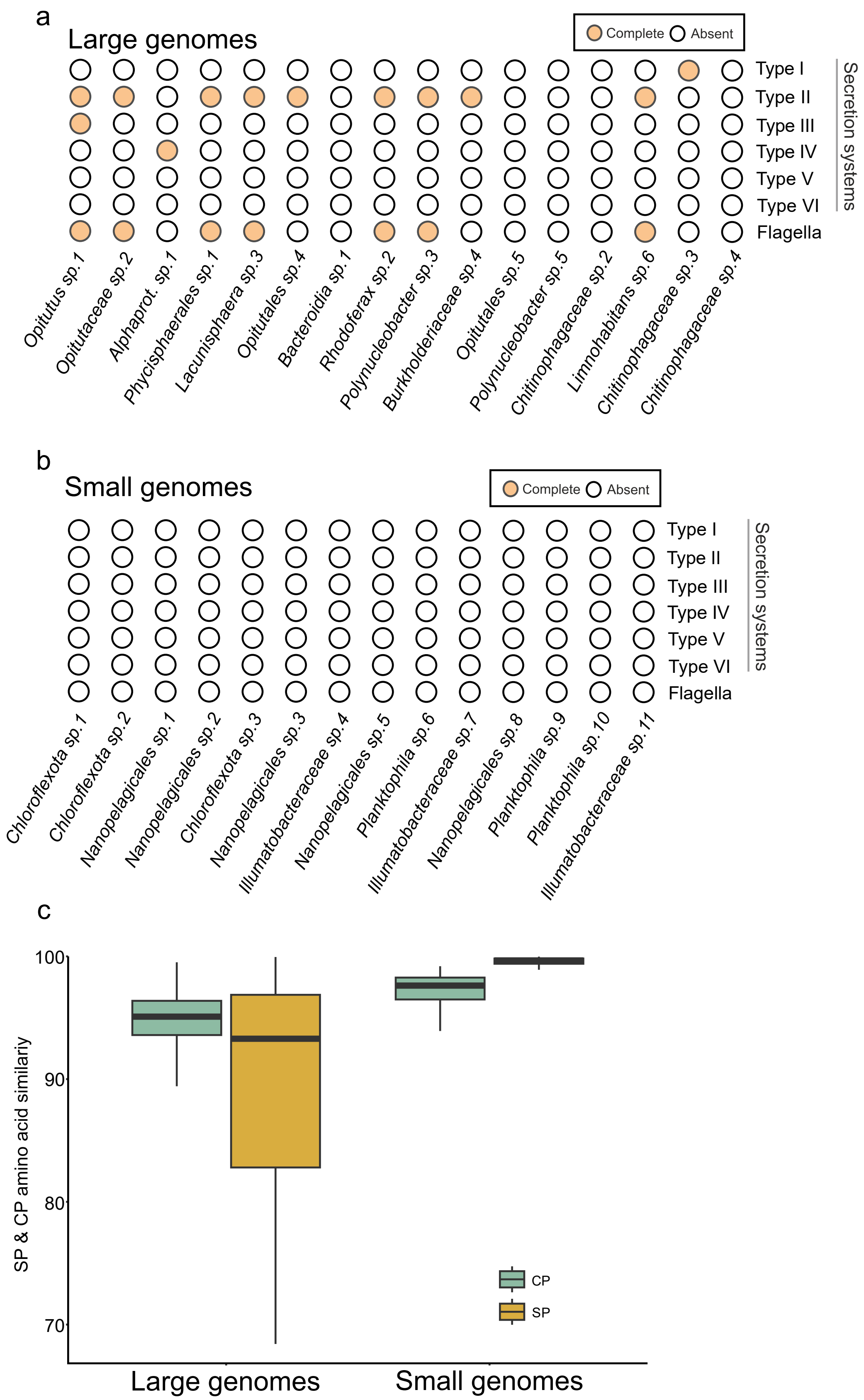

Supplementary Figure S7. Census of metabolic pathways responsible for secretion systems and flagella formation. The figure is separated into two panels representing the absence (empty circle) or presence (orange-filled circle) of secretion systems and flagella (panels a-b). The upper panel focuses on large genome species, while the bottom panel

b) on small genome species. Panel c) illustrates proteome similarities within the secreted proteome (SP) across common proteins between both large and small genome species (Large CP n = 746, SP n = 746; Small CP n = 352, SP n = 350). The central line across the boxplots shows the median, marking the dataset's midpoint. The box itself demarcates the interquartile range, extending from the first quartile to the third quartile, encapsulating the central 50% of the data. The whiskers project from the box to the furthest data points not categorized as outliers and show the spread of the main body of the dataset. Raw data is provided as a Source Data file.

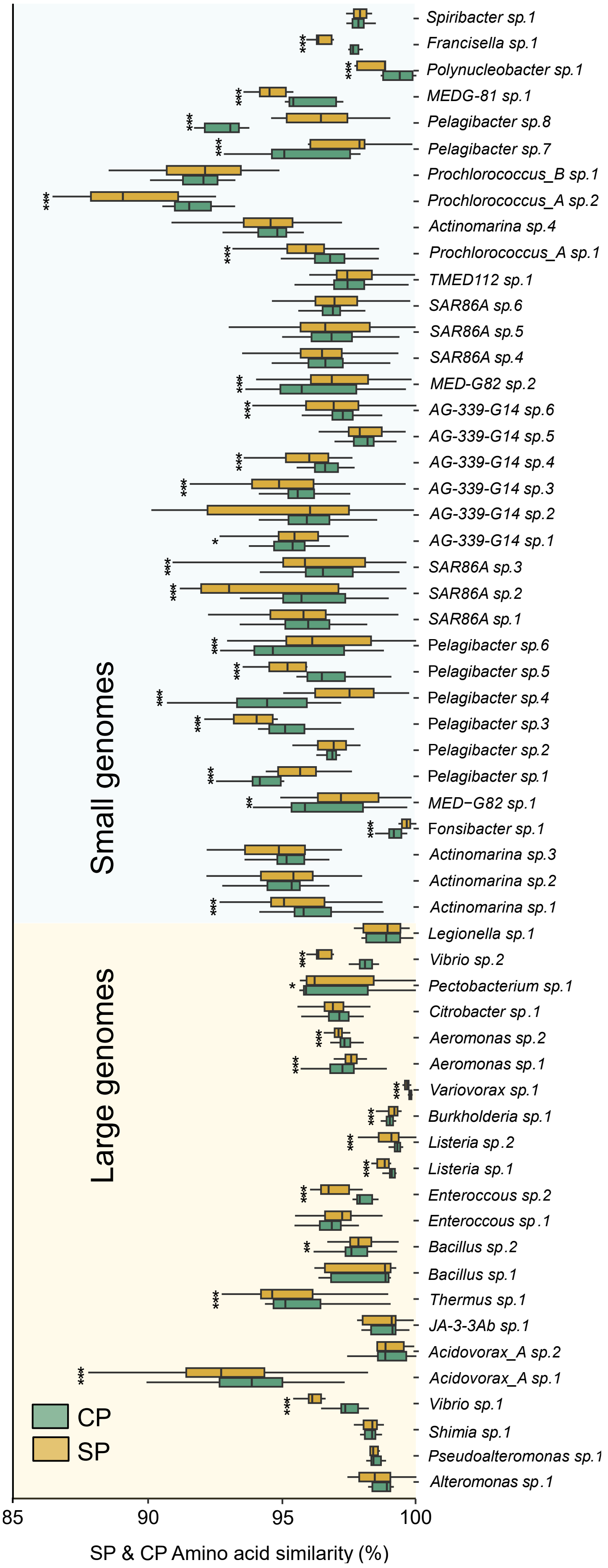

Spiribacter sp.1
Francisella sp.1
Polynucleobacter sp.1
MEDG-81 sp.1
Pelagibacter sp.8
Pelagibacter sp.7
Prochlorococcus_B sp.1
Prochlorococcus_A sp.2
Actinomarina sp.4
Prochlorococcus_A sp.1
TMED112 sp.1
SAR86A sp.6
SAR86A sp.5
SAR86A sp.4
MED-G82 sp.2
AG-339-G14 sp.6
AG-339-G14 sp.5
AG-339-G14 sp.4
AG-339-G14 sp.3
AG-339-G14 sp.2
AG-339-G14 sp.1
SAR86A sp.3
SAR86A sp.2
SAR86A sp.1
Pelagibacter sp.6
Pelagibacter sp.5
Pelagibacter sp.4
Pelagibacter sp.3
Pelagibacter sp.2
Pelagibacter sp.1
MED−G82 sp.1
Fonsibacter sp.1
Actinomarina sp.3
Actinomarina sp.2
Actinomarina sp.1
Legionella sp.1
Vibrio sp.2
Pectobacterium sp.1
Citrobacter sp.1
Aeromonas sp.2
Aeromonas sp.1
Variovorax sp.1
Burkholderia sp.1
Listeria sp.2
Listeria sp.1
Enteroccous sp.2
Enteroccous sp.1
Bacillus sp.2
Bacillus sp.1
Thermus sp.1
JA-3-3Ab sp.1
Acidovorax_A sp.2
Acidovorax_A sp.1
Vibrio sp.1
Shimia sp.1
Pseudoalteromonas sp.1
Alteromonas sp.1
Small genomes
Large genomes
CP
SP
85
90
95
100
SP & CP Amino acid similarity (%)

Supplementary Figure S8. Proteome similarity within species-level boundaries of the extended dataset. The figure depicts the within-species identities of secreted (SP) and cytoplasmic (CP) proteomes within an extended environmental dataset (*AG-339-G14 sp.1* CP n = 242, SP n = 242; *AG-339-G14 sp.2* CP n = 110, SP n = 110; *AG-339-G14 sp.3* CP n = 156, SP n = 156; *AG-339-G14 sp.4* CP n = 90, SP n = 90; *AG-339-G14 sp.5* CP n = 90, SP n = 90; *AG-339-G14 sp.6* CP n = 420, SP n = 418; *Acidovorax_A sp.1* CP n = 306, SP n = 306; *Acidovorax_A sp.2* CP n = 240, SP n = 236; *Actinomarina sp.1* CP n = 86, SP n = 86; *Actinomarina sp.2* CP n = 88, SP n = 88; *Actinomarina sp.3* CP n = 104, SP n = 104; *Actinomarina sp.4* CP n = 70, SP n = 70; *Aeromonas sp.1* CP n = 108, SP n = 110; *Aeromonas sp.2* CP n = 110, SP n = 110; *Alteromonas sp.1* CP n = 36, SP n = 40; *Bacillus sp.1* CP n = 30, SP n = 30; *Bacillus sp.2* CP n = 156, SP n = 152; *Burkholderia sp.1* CP n = 90, SP n = 86; *Citrobacter sp.1* CP n = 56, SP n = 56; *Enterococcus sp.1* CP n = 56, SP n = 56; *Enterococcus sp.2* CP n = 30, SP n = 30; *Fonsibacter sp.1* CP n = 55, SP n = 55; *Francisella sp.1* CP n = 72, SP n = 70; JA-3-3Ab sp 1 CP n = 30, SP n = 30; *Legionella sp.1* CP n = 72, SP n = 72; *Listeria sp.1* CP n = 108, SP n = 110; *Listeria sp.2* CP n = 110, SP n = 110; *MED-G82 sp.1* CP n = 45, SP n = 45; *MED-G82 sp.2* CP n = 182, SP n = 182; *MEDG-81 sp.1* CP n = 20, SP n = 20; *Pectobacterium sp.1* CP n = 42, SP n = 42; *Pelagibacter sp.1* CP n = 42, SP n = 42; *Pelagibacter sp.2* CP n = 42, SP n = 42; *Pelagibacter sp.3* CP n = 42, SP n = 42; *Pelagibacter sp.4* CP n = 42, SP n = 42; *Pelagibacter sp.5* CP n = 42, SP n = 42; *Pelagibacter sp.6* CP n = 72, SP n = 72; *Pelagibacter sp.7* CP n = 28, SP n = 28; *Pelagibacter sp.8* CP n = 20, SP n = 20; *Polynucleobacter sp.1* CP n = 40, SP n = 28; *Prochlorococcus_A sp.1* CP n = 1056, SP n = 1056; *Prochlorococcus_A sp.2* CP n = 30, SP n = 30; *Prochlorococcus_B sp.1* CP n = 30, SP n = 30; *Pseudoalteromonas sp.1* CP n = 34, SP n = 34; *SAR86A sp.1* CP n = 240, SP n = 240; *SAR86A sp.2* CP n = 56, SP n = 56; *SAR86A sp.3* CP n = 90, SP n = 90; *SAR86A sp.4* CP n = 420, SP n = 420; *SAR86A sp.5* CP n = 272, SP n = 272; *SAR86A sp.6* CP n = 110, SP n = 110; *Shimia sp.1* CP n = 72, SP n = 72; *Spiribacter sp.1* CP n = 42, SP n = 40; *TMED112 sp.1* CP n = 72, SP n = 72; *Thermus sp.1* CP n = 182, SP n = 182; *Variovorax sp.1* CP n = 30, SP n = 30; *Vibrio sp.1* CP n = 70, SP n = 66; *Vibrio sp.2* CP n = 156, SP n = 70;). Statistical differences are highlighted through stars and obtained by Wilcox test (*Actinomarina sp.1* p-value = 2.19235713095448e-05, *Actinomarina sp.2* p-value = 0.237115285, *Actinomarina sp.3* p-value = 0.009356837, *Fonsibacter sp.1* p-value = 5.94954438992676e-13, *MED-G82 sp.1* p-value = 0.004735963, *Pelagibacter sp.1* p-value = 2.04026020411072e-08, *Pelagibacter sp.2* p-value = 0.861411809, *Pelagibacter sp.3* p-value = 1.07774708360947e-05, *Pelagibacter sp.4* p-value = 3.86574446219976e-10, *Pelagibacter sp.5* p-value = 5.12653343288079e-06, *Pelagibacter sp.6* p-value = 0.000110558, *SAR86A sp.1* p-value = 0.013400024, *SAR86A sp.2* p-value = 0.002223998, *SAR86A sp.3* p-value = 0.084220247, *SAR86A sp.4* p-value = 0.072008007, *SAR86A sp.5* p-value = 0.282130779, *SAR86A sp.6* p-value = 0.436231607, *TMED112 sp.1* p-value = 0.277927713, *Alteromonas sp.1* p-value = 0.067455604, *Pseudoalteromonas sp.1* p-value = 0.591255954, *Shimia sp.1* p-value = 0.582583602, *Vibrio sp.1* p-value = 4.01004240451295e-17, *Acidovorax A sp.1* p-value = 1.93524475592149e-10, *Acidovorax A sp.2* p-value = 0.064523232, *Prochlorococcus A sp.1* p-value = 8.86223554672511e-97, *Actinomarina sp.4* p-value = 0.306103665, *Prochlorococcus A sp.2* p-value = 0.000123745, *Prochlorococcus B sp.1* p-value = 0.864923062, *Pelagibacter sp.7* p-value = 1.12212409059951e-05, *Pelagibacter sp.8* p-value = 6.53186699717988e-08, *MEDG 81 sp.1* p-value = 0.000243758, *JA-3-3Ab sp.1* p-value = 0.620219045, *Thermus sp.1* p-value = 2.56445125875593e-08, *Bacillus sp.1* p-value = 0.750325529, *AG-339-G14 sp.1* p-value = 0.01774028, *Bacillus sp.2* p-value =

0.000385577, *Enterococcus sp.1* p-value = 0.008883872, *Enterococcus sp.2* p-value = 2.34235944824938e-07, *Listeria sp.1* p-value = 2.10665289985e-13, *Listeria sp.2* p-value = 5.58390582793893e-08, *Burkholderia sp.1* p-value = 9.55873581787196e-05, *Polynucleobacter sp.1* p-value = 4.26171331967336e-06, *Variovorax sp.1* p-value = 4.24248645230498e-07, *Aeromonas sp.1* p-value = 7.18166888414335e-07, *Aeromonas sp.2* p-value = 4.46422734425325e-10, AG-339-G14 sp.2 p-value = 0.367371131, *Citrobacter sp.1* p-value = 0.15639099, *Pectobacterium sp.1* p-value = 0.035007574, *Vibrio sp.2* p-value = 4.88322201118117e-18, *Francisella sp.1* p-value = 6.33181591372013e-15, *Legionella sp.1* p-value = 0.48800807, *AG-339-G14 sp.3* p-value = 2.99155872372462e-05, AG-339-G14 sp.4 p-value = 3.05167672700752e-07, *AG-339-G14 sp.5* p-value = 0.736718269, *AG-339-G14 sp.6* p-value = 1.59987119807954e-05, *Spiribacter sp.1* p-value = 4.47233707801023e-08). The central line across the boxplots shows the median, marking the dataset's midpoint. The box itself demarcates the interquartile range, extending from the first quartile to the third quartile, encapsulating the central 50% of the data. The whiskers project from the box to the furthest data points not categorized as outliers and show the spread of the main body of the dataset. Raw data is provided as a Source Data file.

Alphaprot. sp.1

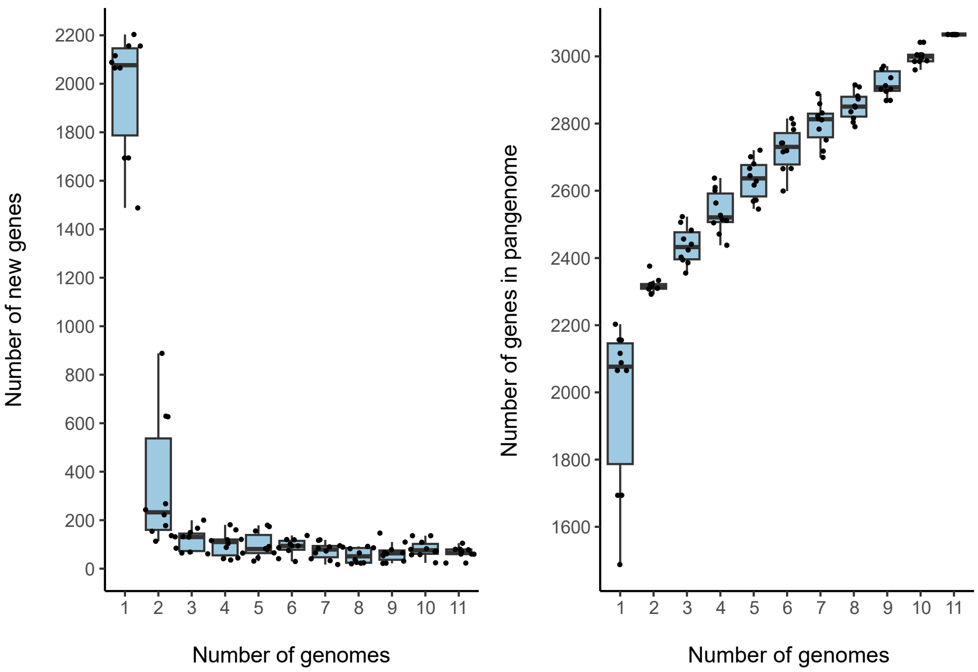

Bacteroidia sp.1

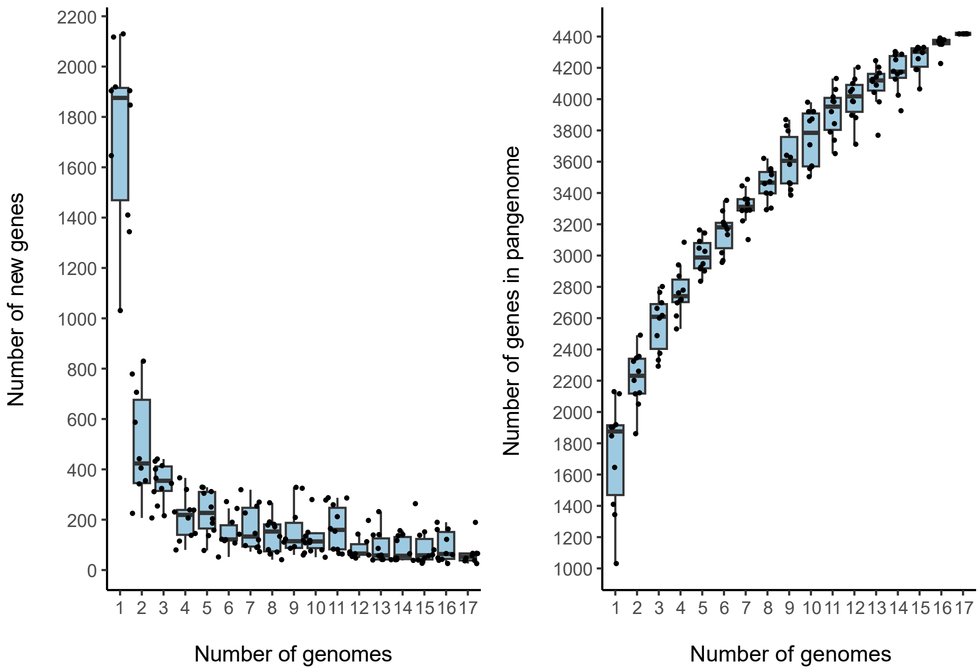

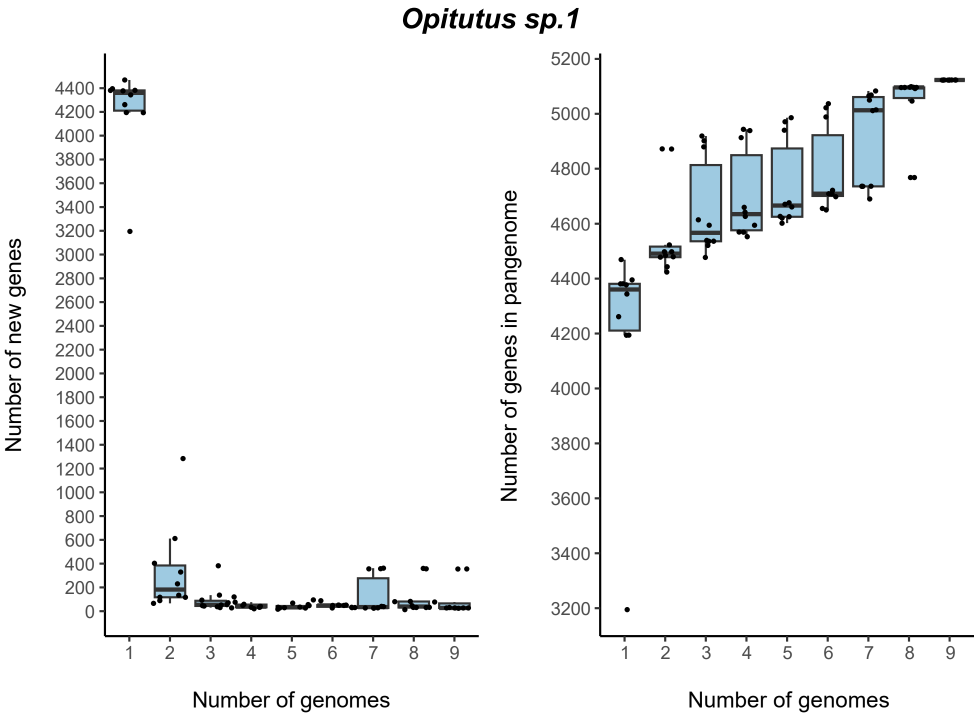
Opitutus sp.1
Number of new genes
Number of genes in pangenome
Number of genomes
Number of genomes

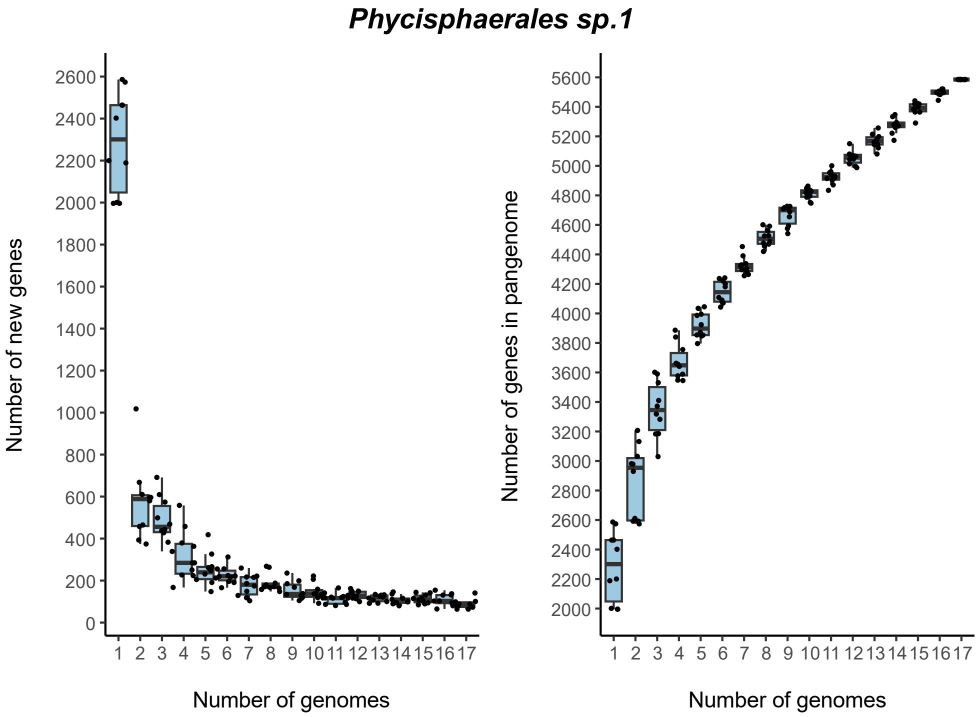
Phycisphaerales sp.1
Number of new genes
Number of genes in pangenome
Number of genomes
Number of genomes

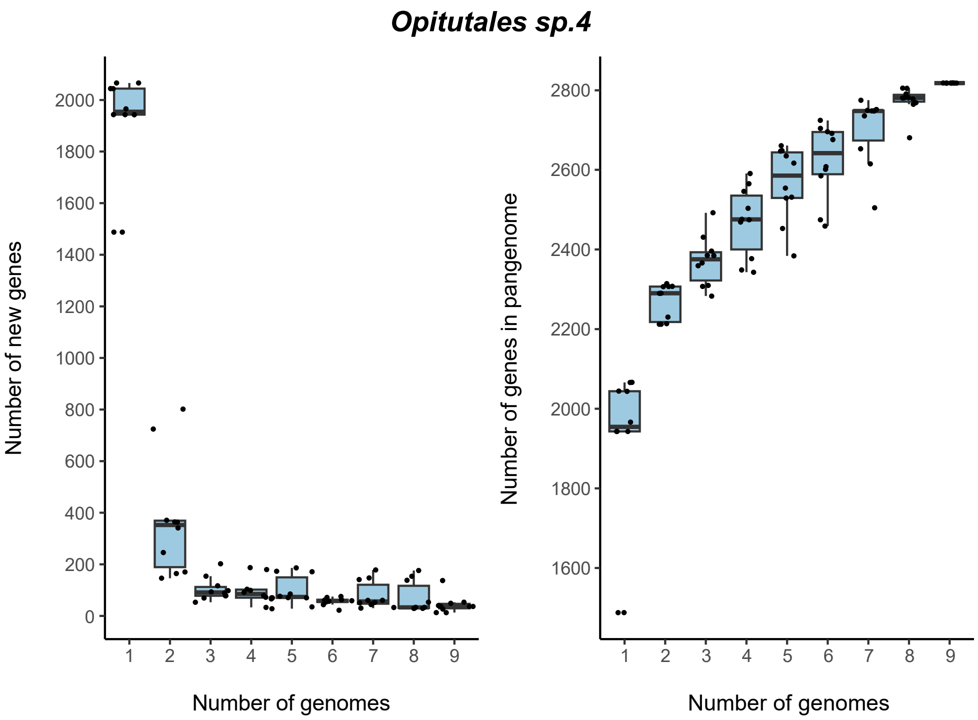
Opitutales sp.4
Number of new genes
Number of genes in pangenome
Number of genomes
Number of genomes

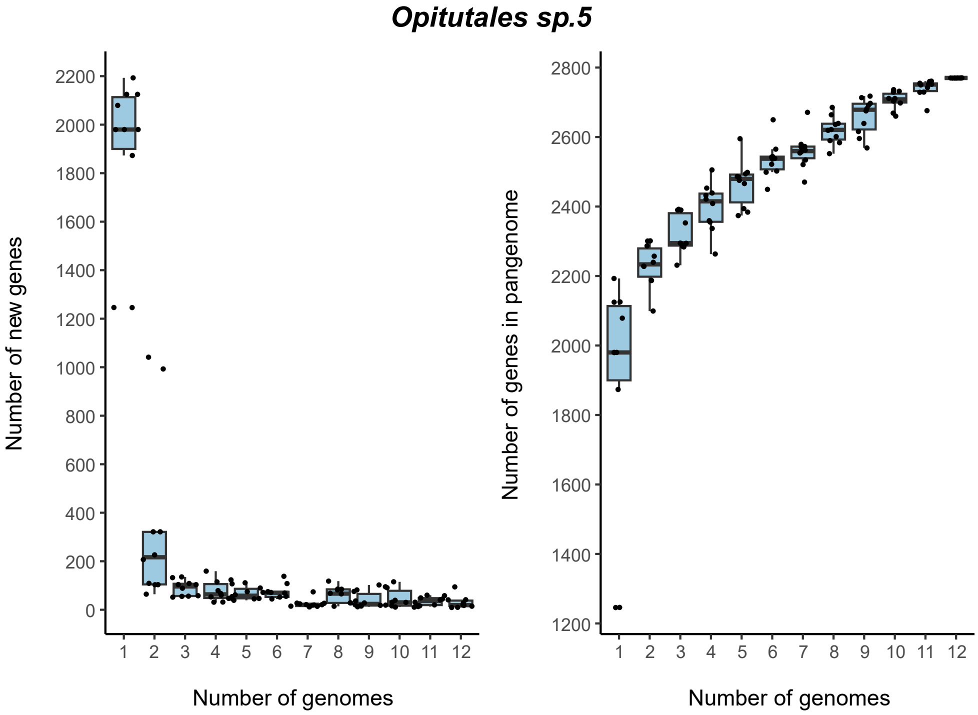
Opitutales sp.5
Number of new genes
Number of genes in pangenome
Number of genomes
Number of genomes

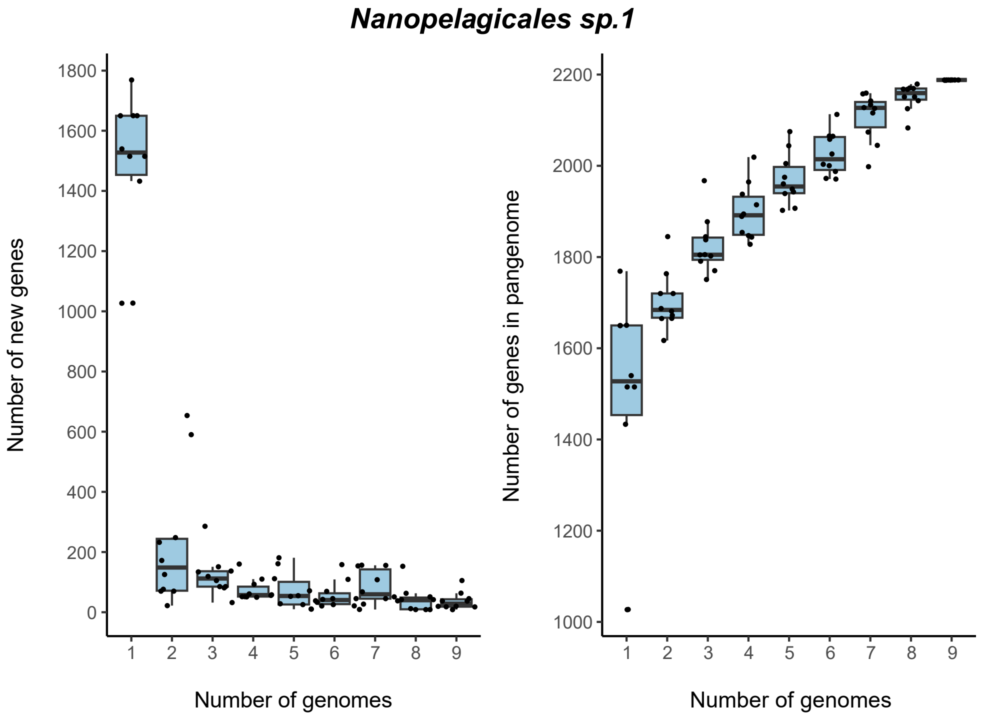
Nanopelagicales sp.1
Number of new genes
0
200
400
600
800
1000
1200
1400
1600
1800
Number of genomes
1 2 3 4 5 6 7 8 9
Number of genes in pangenome
1000
1200
1400
1600
1800
2000
2200
Number of genomes
1 2 3 4 5 6 7 8 9

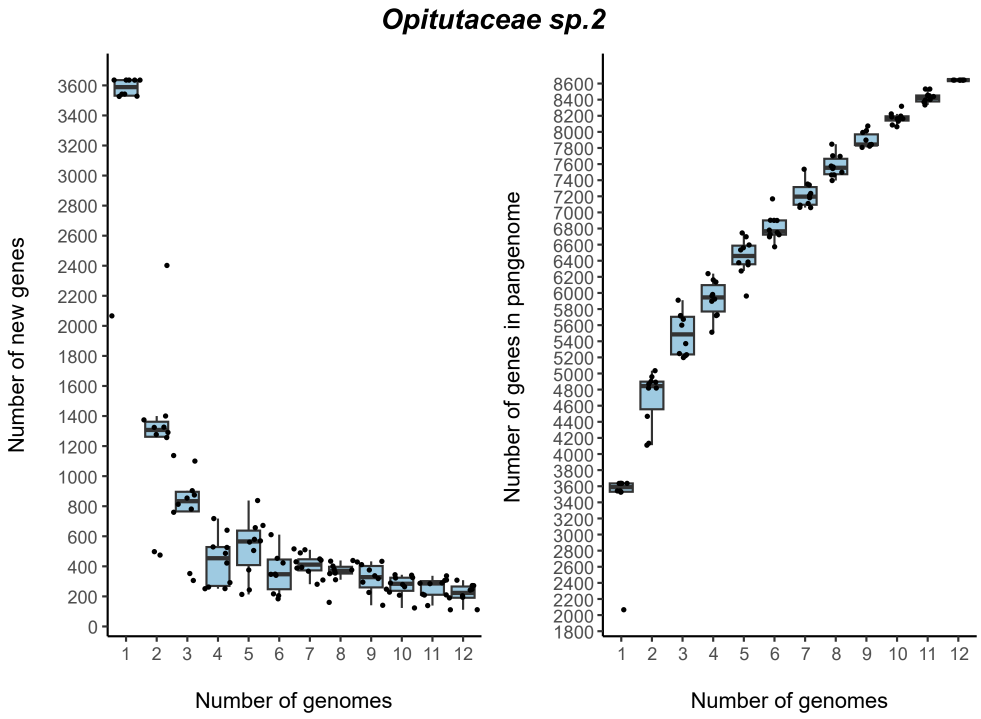
Opitutaceae sp.2
Number of new genes
0
200
400
600
800
1000
1200
1400
1600
1800
2000
2200
2400
2600
2800
3000
3200
3400
3600
Number of genomes
1 2 3 4 5 6 7 8 9 10 11 12
Number of genes in pangenome
1800
2000
2200
2400
2600
2800
3000
3200
3400
3600
3800
4000
4200
4400
4600
4800
5000
5200
5400
5600
5800
6000
6200
6400
6600
6800
7000
7200
7400
7600
7800
8000
8200
8400
8600
Number of genomes
1 2 3 4 5 6 7 8 9 10 11 12

Nanopelagicaceae sp.5

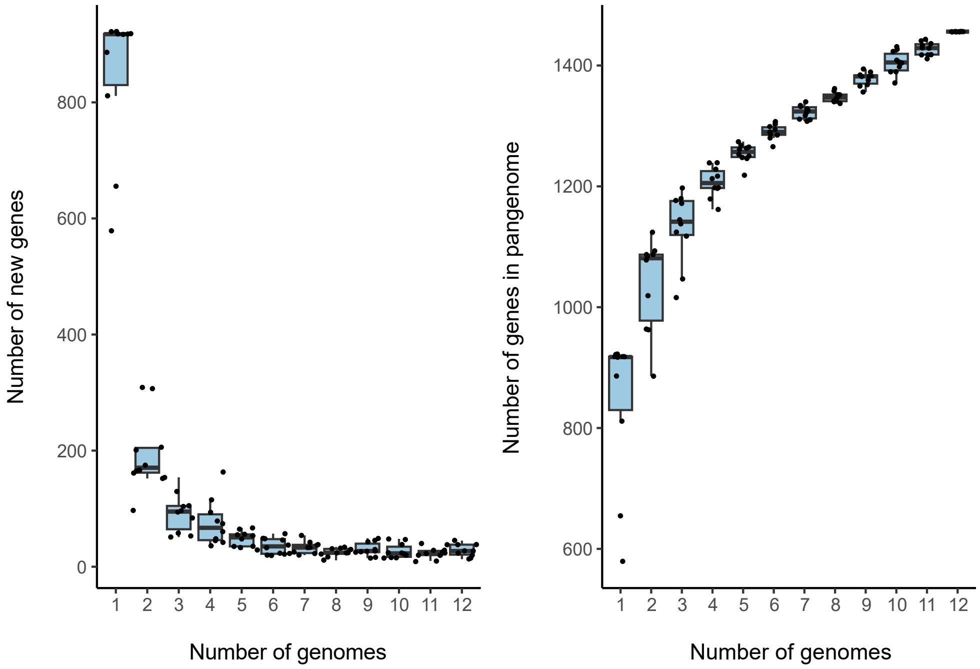

Nanopelagicaceae sp.8

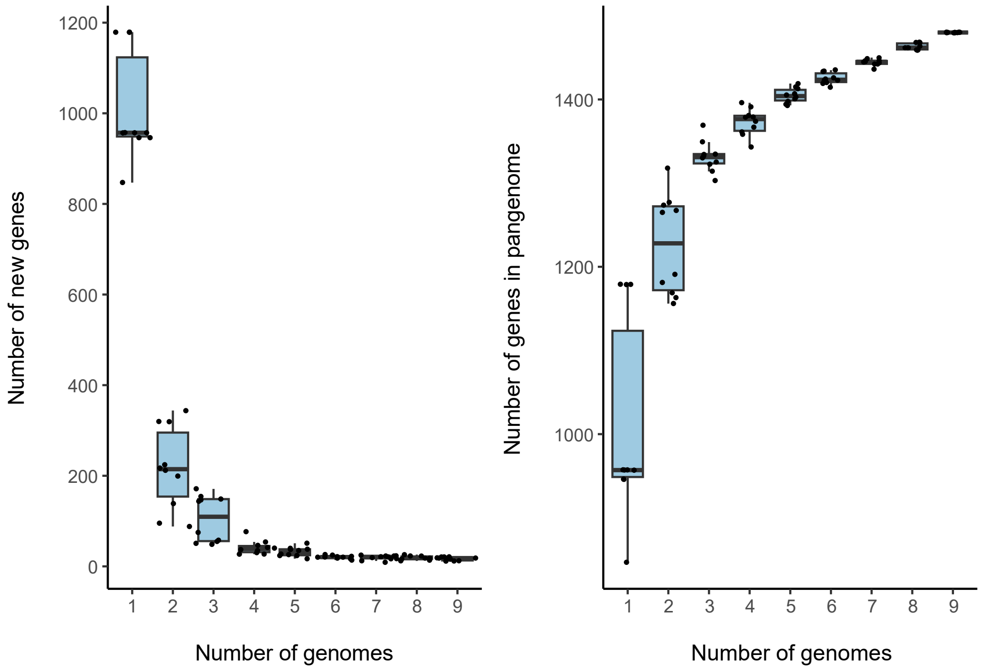

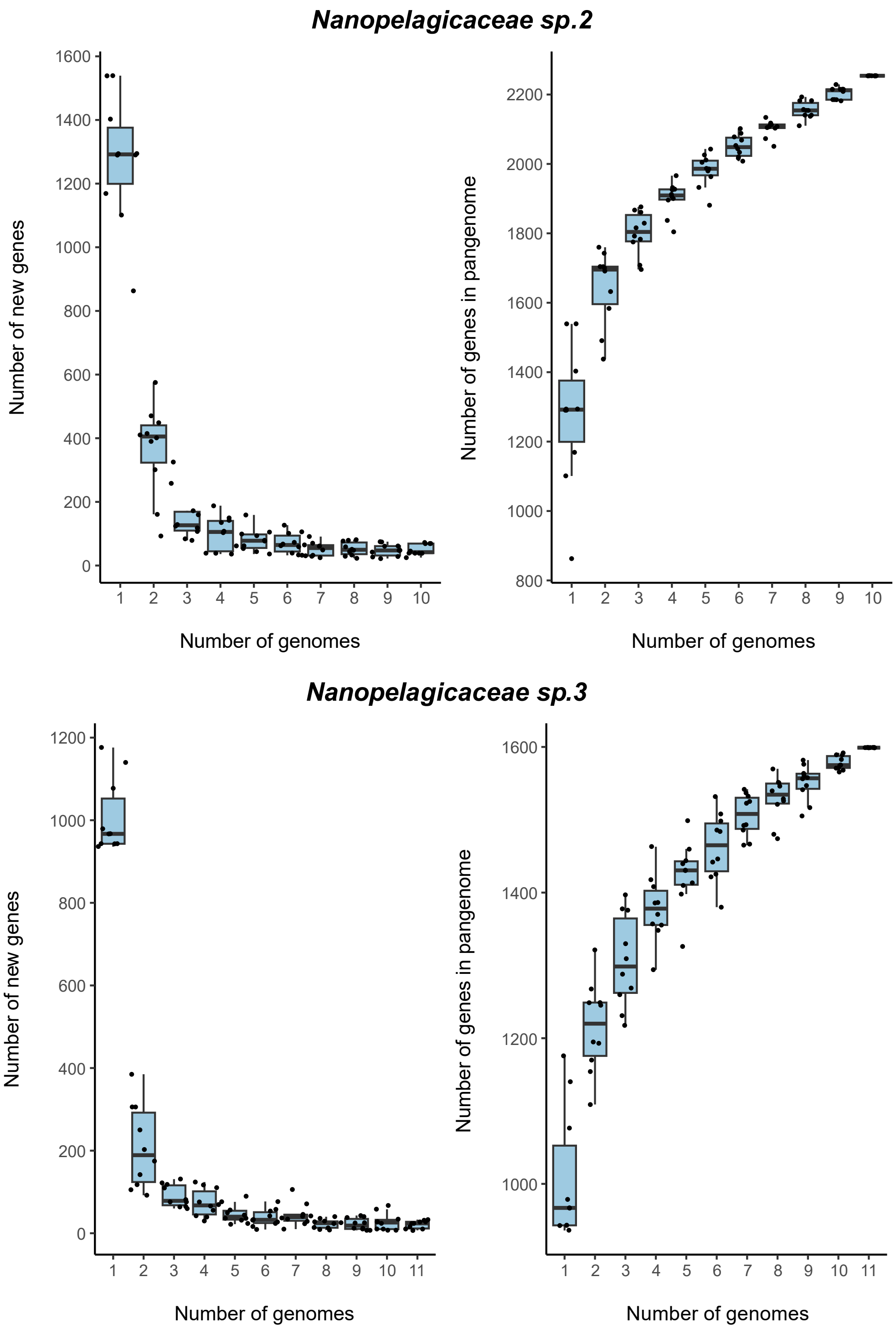
Nanopelagicaceae sp.2
Number of new genes
Number of genes in pangenome
Number of genomes
Number of genomes
Nanopelagicaceae sp.3
Number of new genes
Number of genes in pangenome
Number of genomes
Number of genomes

Ilumatobacteraceae sp.7

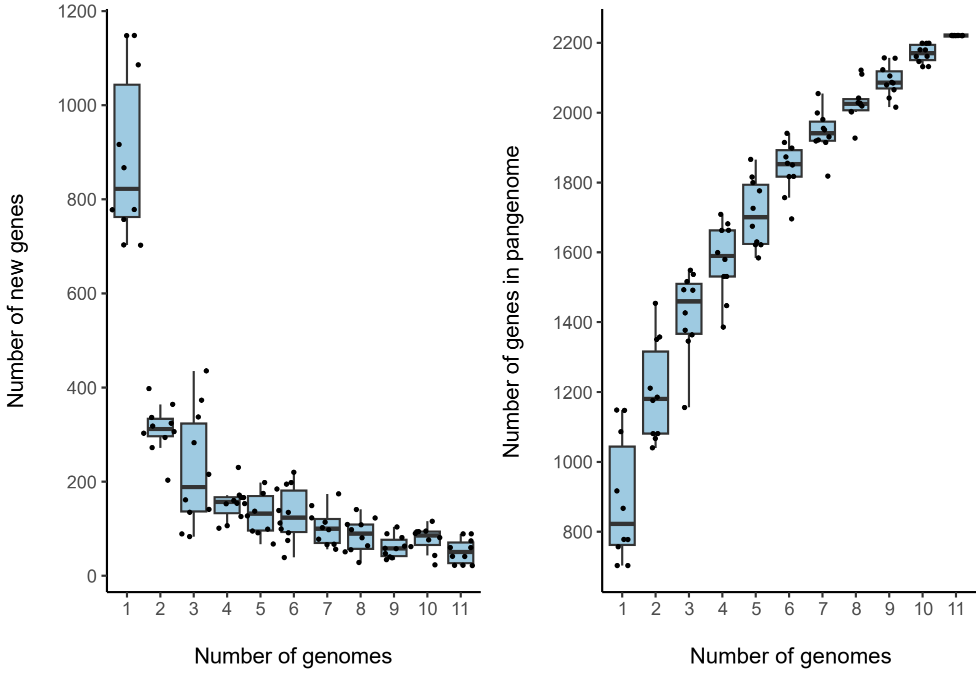

Ilumatobacteraceae sp.11

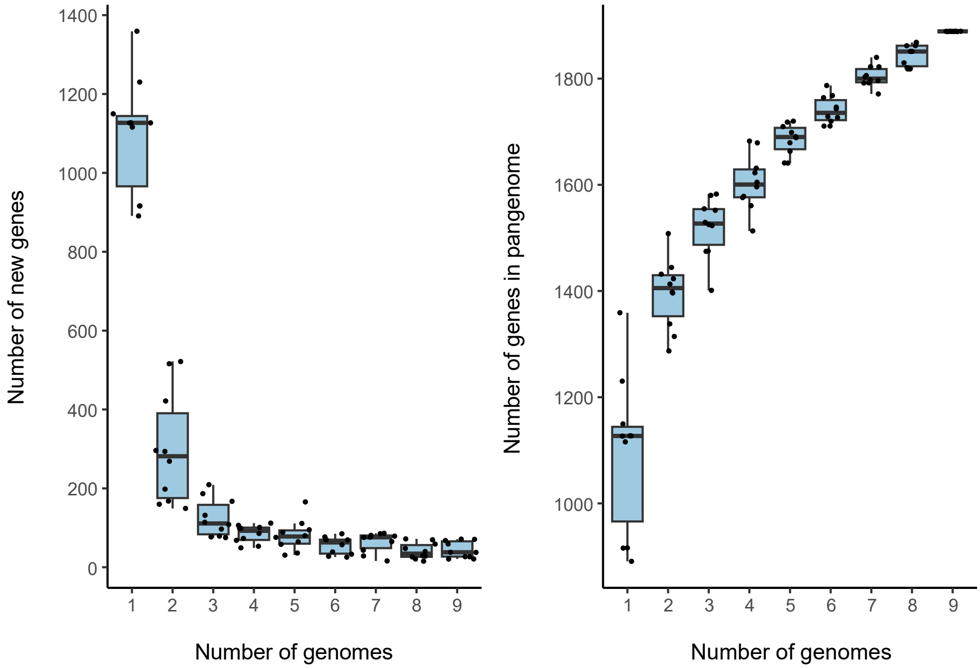

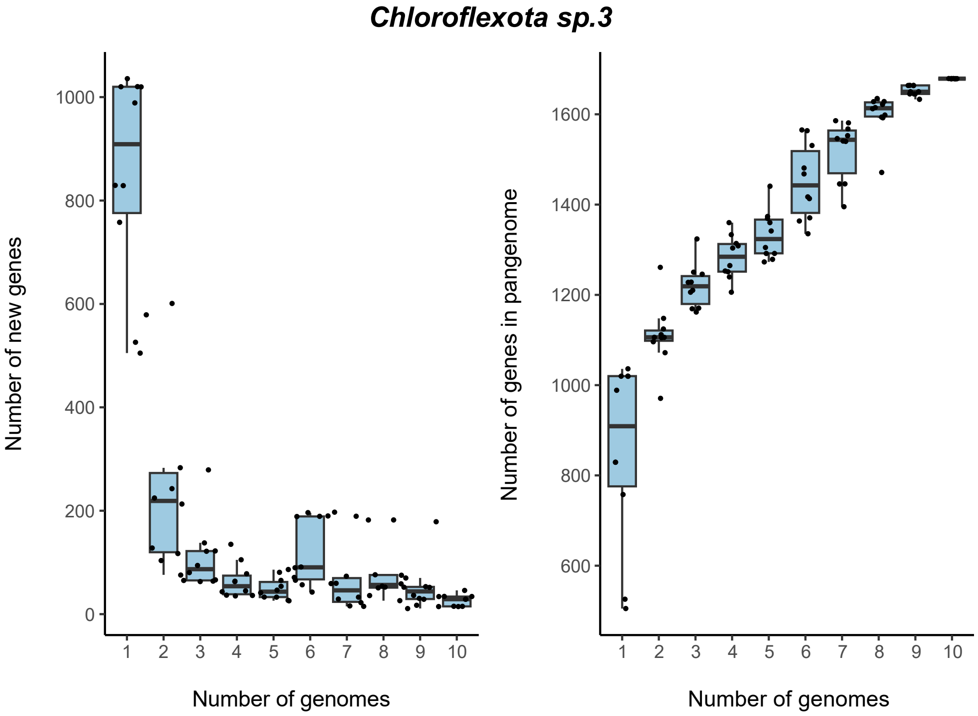

Chloroflexota sp.3
Number of new genes
0
200
400
600
800
1000
Number of genomes
1
2
3
4
5
6
7
8
9
10
Number of genes in pangenome
600
800
1000
1200
1400
1600
Number of genomes

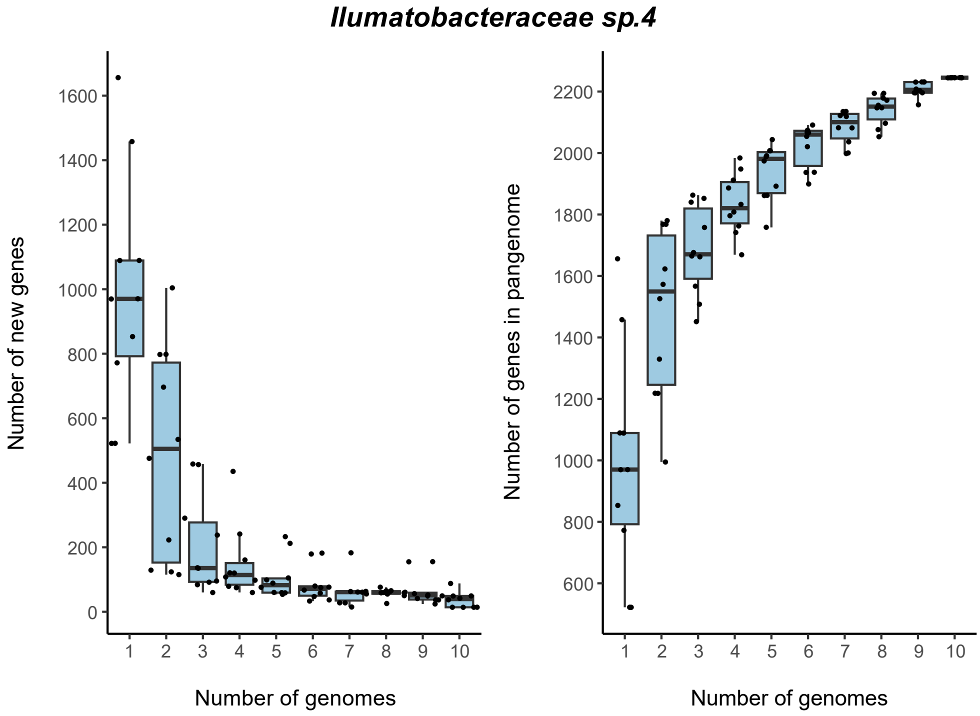

Ilumatobacteraceae sp.4
Number of new genes
0
200
400
600
800
1000
1200
1400
1600
Number of genomes
1
2
3
4
5
6
7
8
9
10
Number of genes in pangenome
600
800
1000
1200
1400
1600
1800
2000
2200
Number of genomes

Chloroflexota sp.1

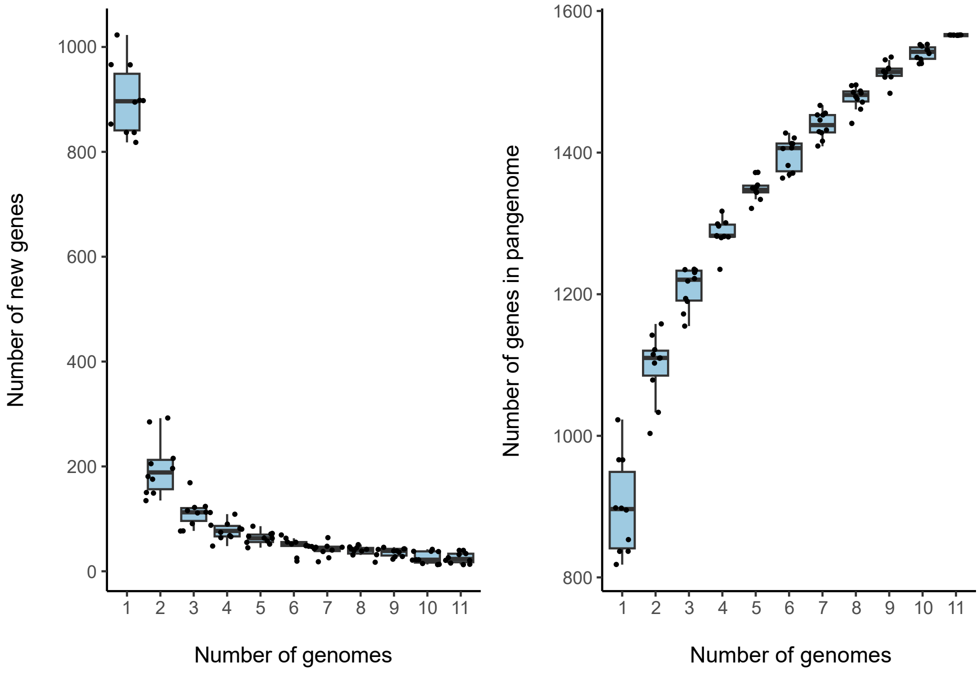

Chloroflexota sp.2

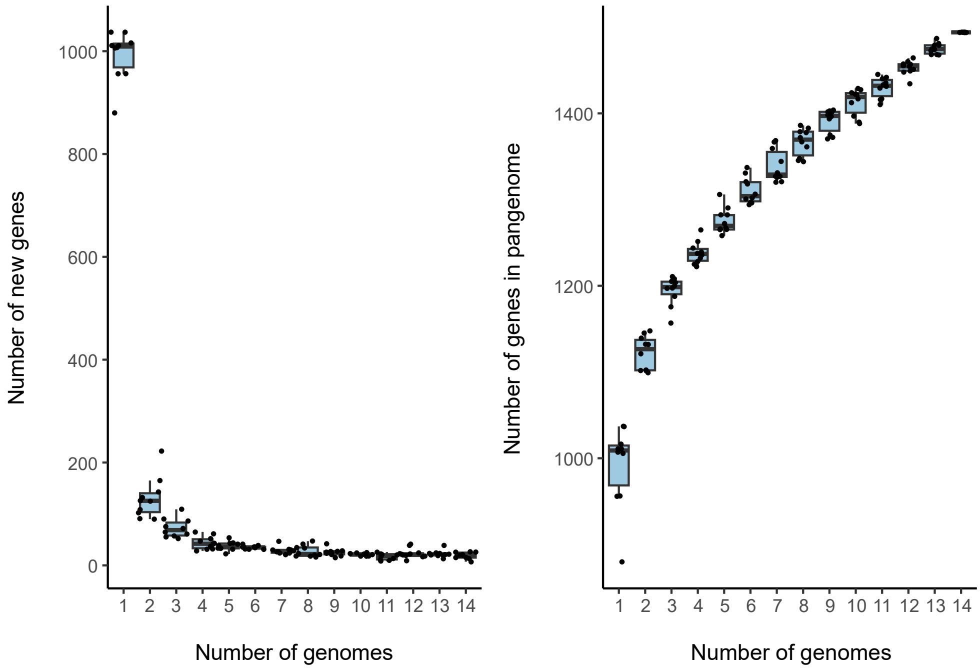

Chitinophagaceae sp.3

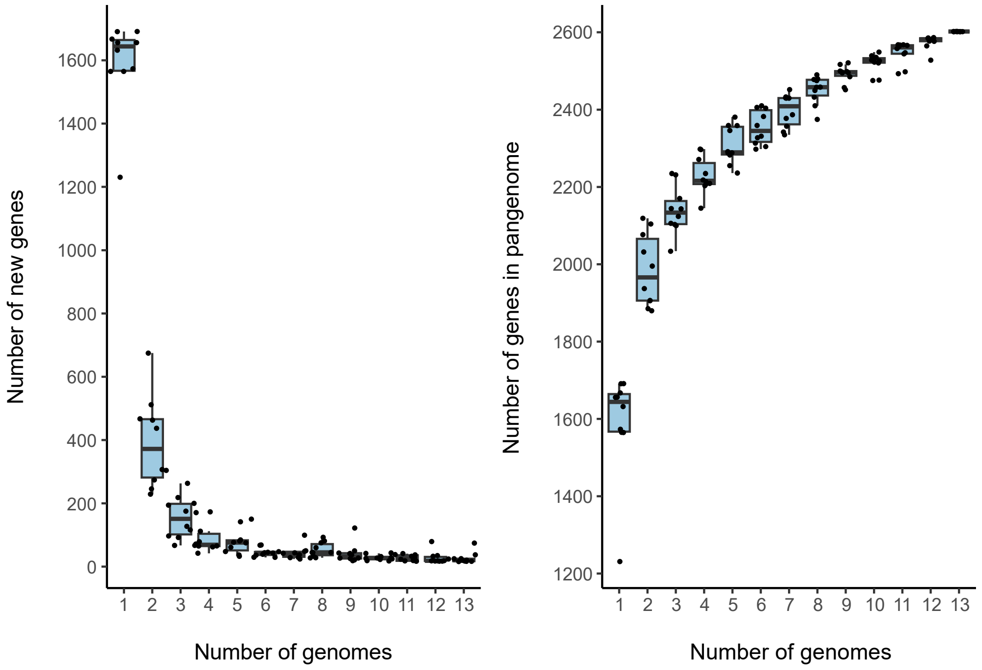

Chitinophagaceae sp.4

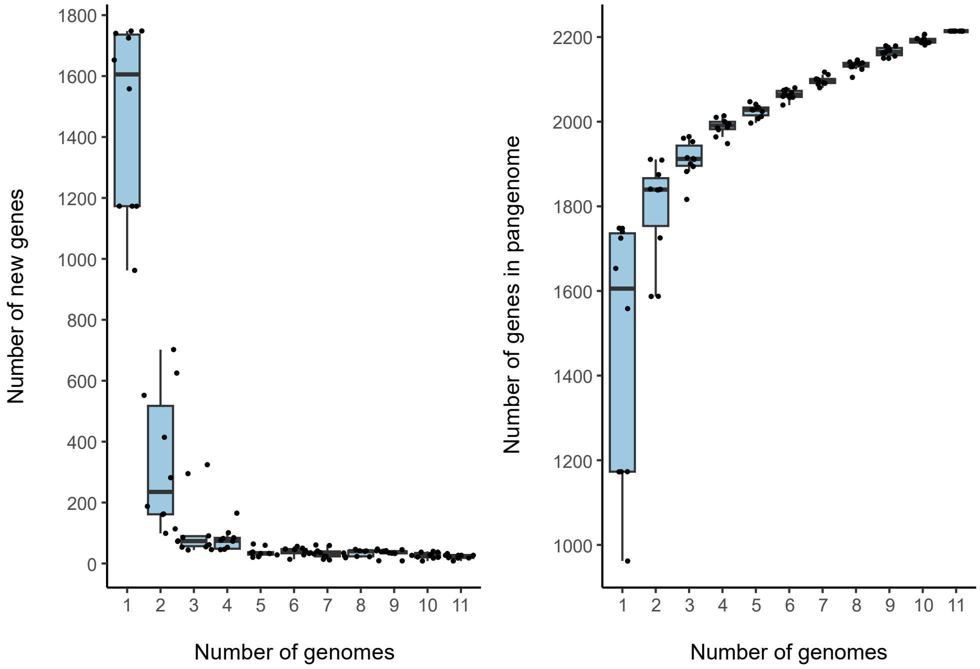

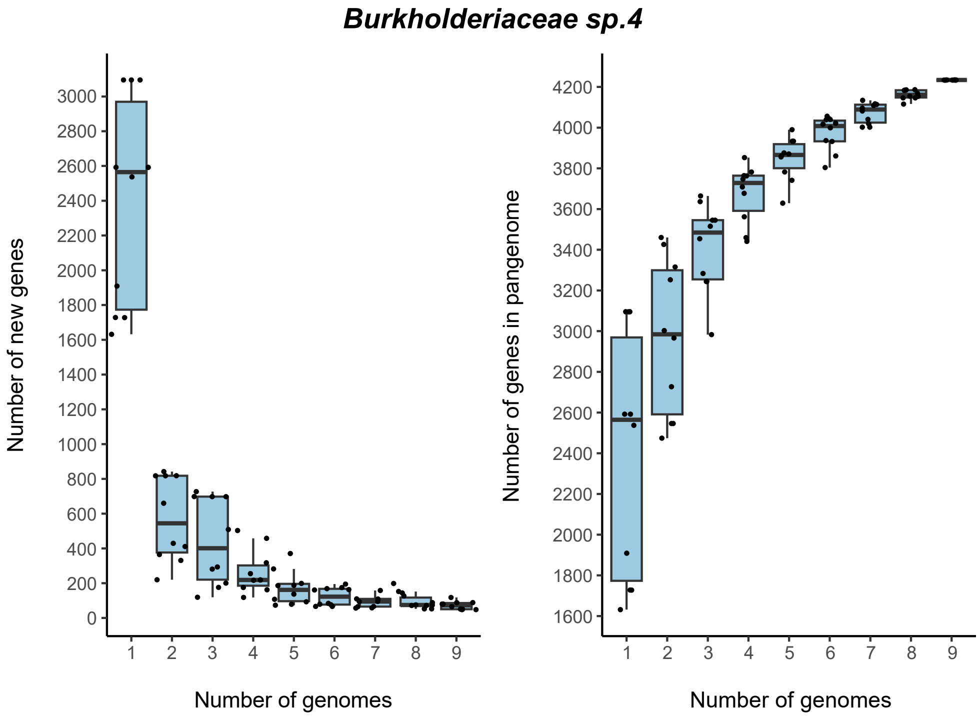
Burkholderiaceae sp.4
Number of new genes
3000
2800
2600
2400
2200
2000
1800
1600
1400
1200
1000
800
600
400
200
0
1 2 3 4 5 6 7 8 9
Number of genomes
Number of genes in pangenome
4200
4000
3800
3600
3400
3200
3000
2800
2600
2400
2200
2000
1800
1600
1 2 3 4 5 6 7 8 9
Number of genomes

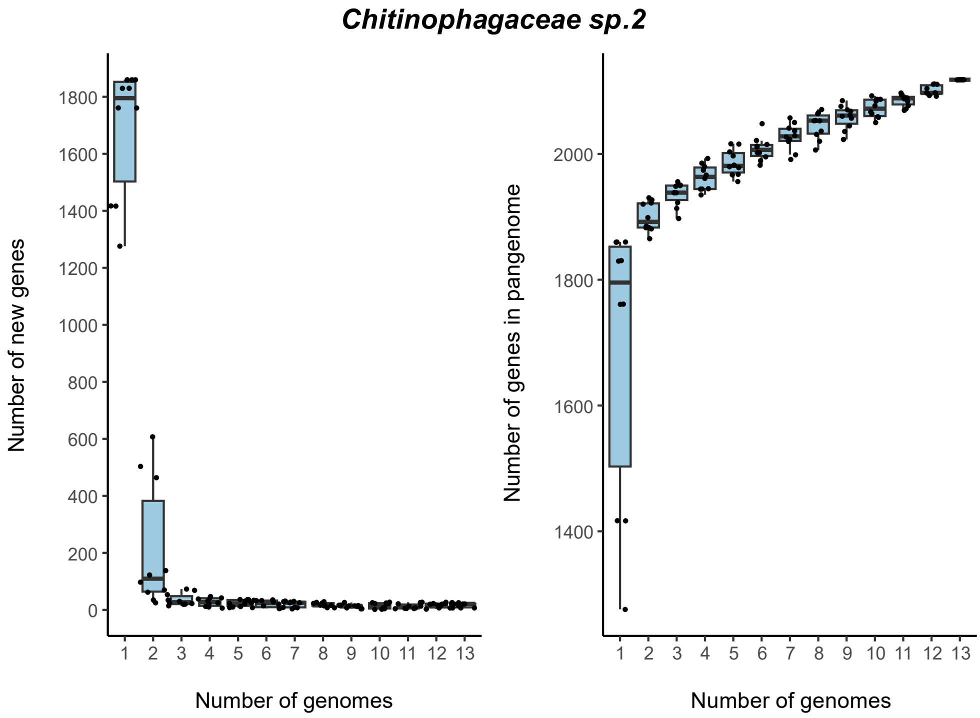
Chitinophagaceae sp.2
Number of new genes
1800
1600
1400
1200
1000
800
600
400
200
0
1 2 3 4 5 6 7 8 9 10 11 12 13
Number of genomes
Number of genes in pangenome
2000
1800
1600
1400
1 2 3 4 5 6 7 8 9 10 11 12 13
Number of genomes

Polynucleobacter sp.5

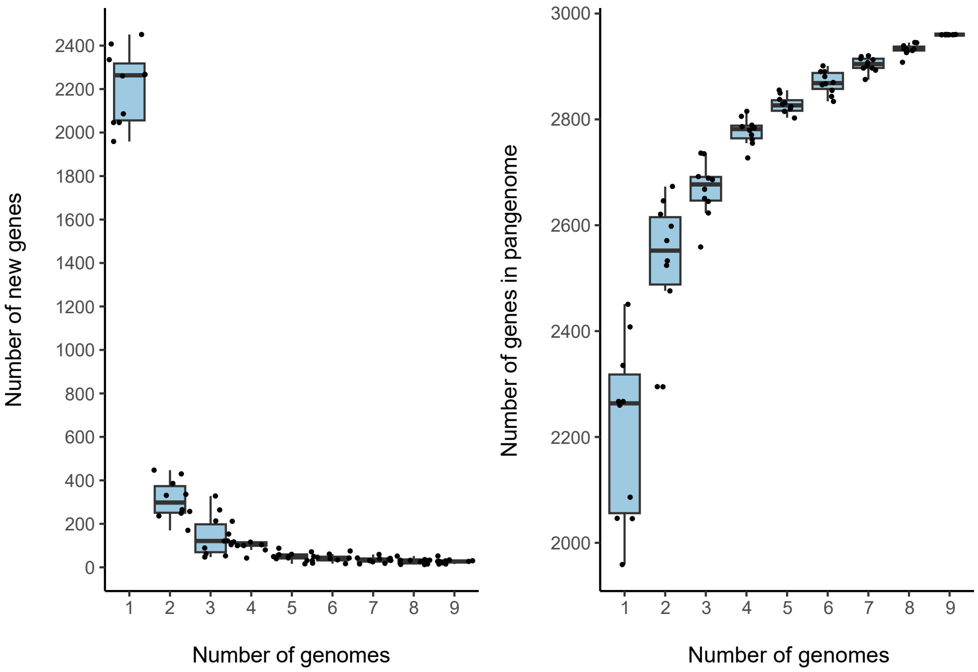

Rhodoferax sp.2

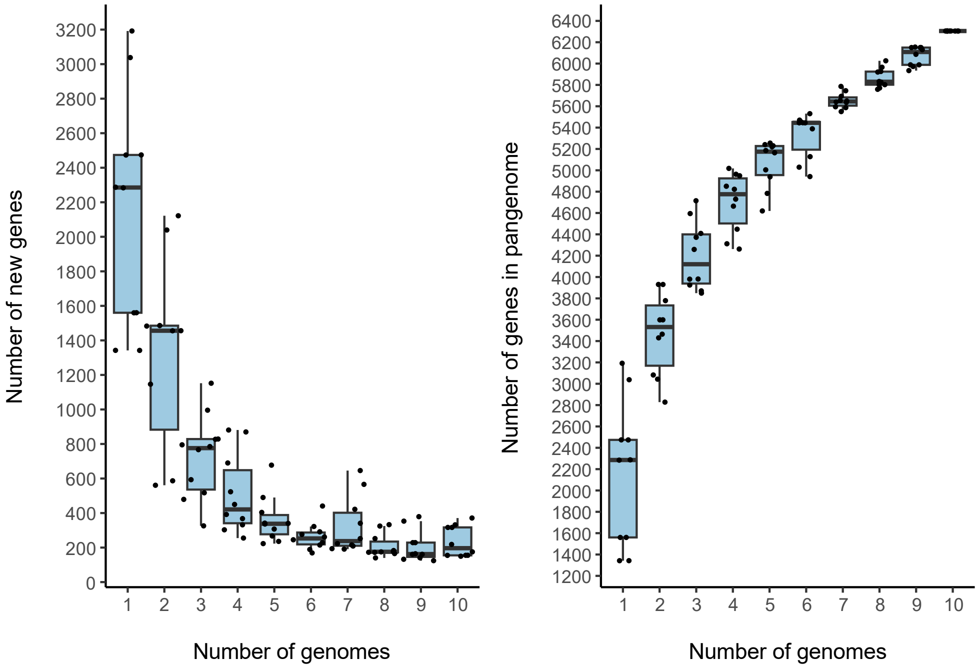

Planktophila sp.10

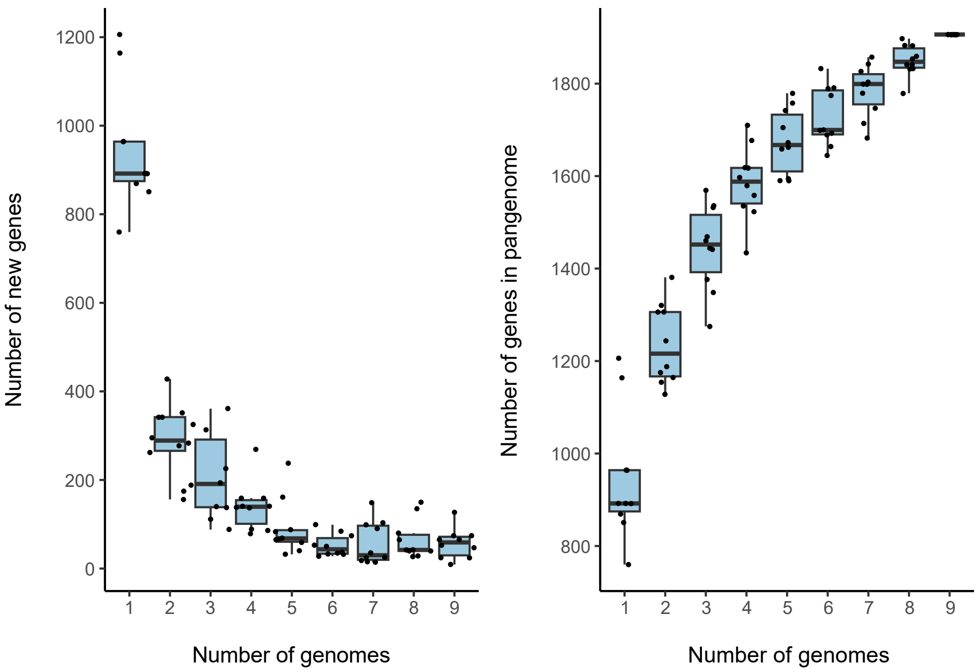

Polynucleobacter sp.3

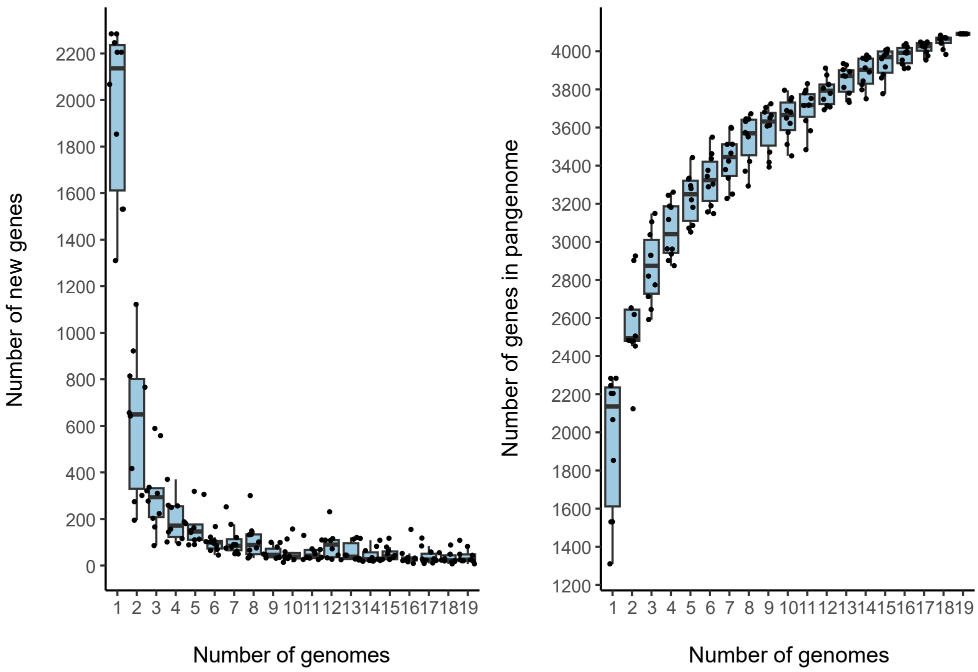

Planktophila sp.6

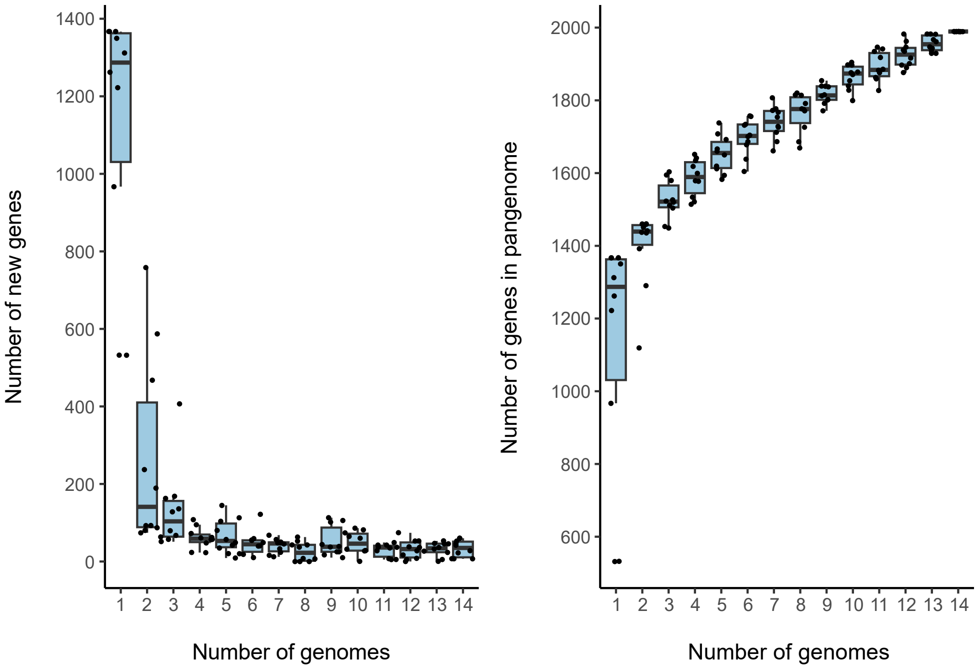

Planktophila sp.9

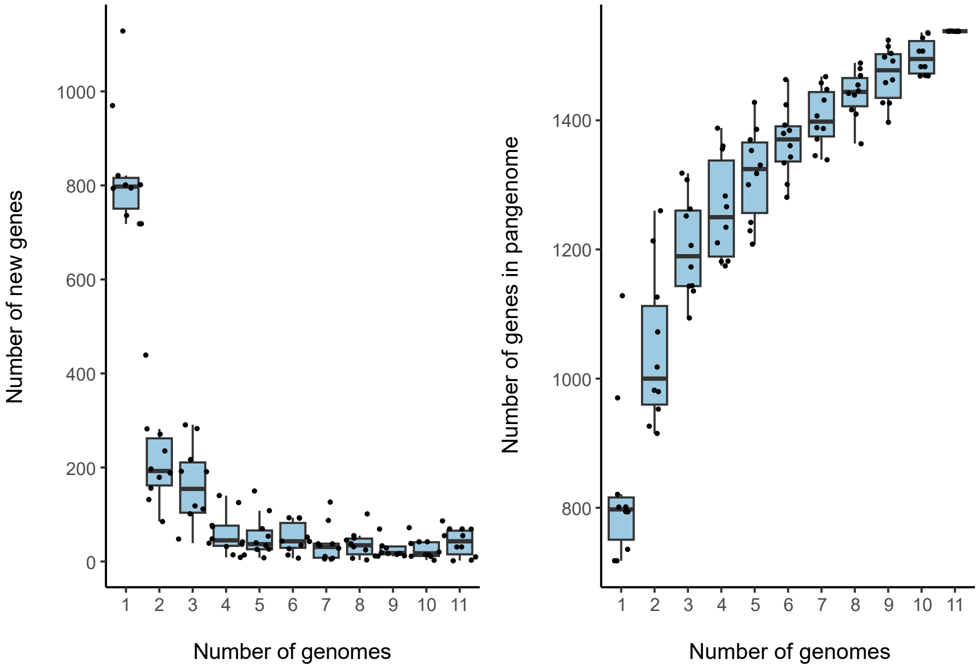

Opitutus sp.1

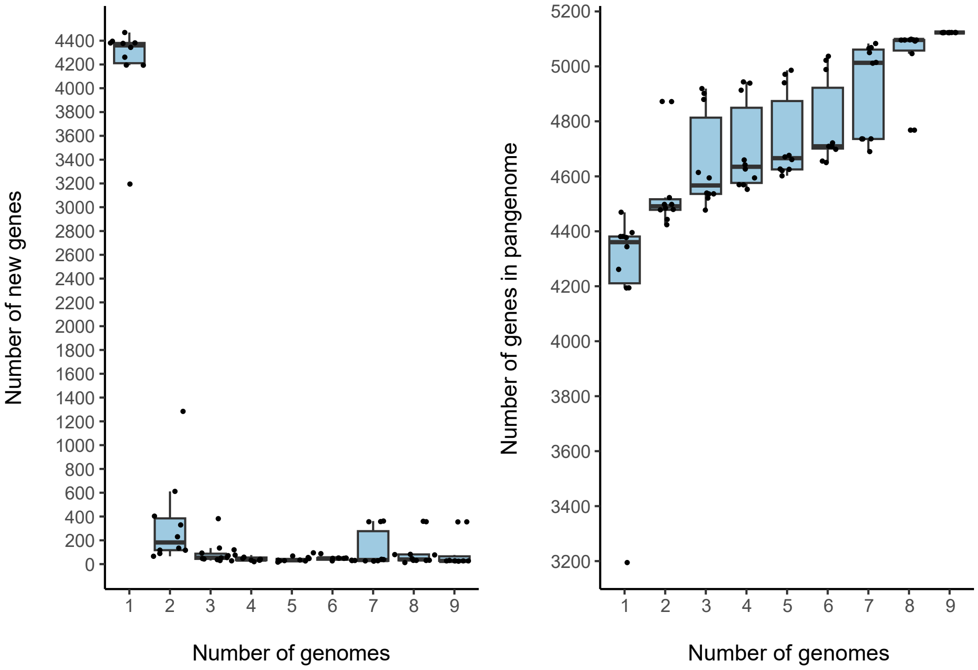

Phycisphaerales sp.1

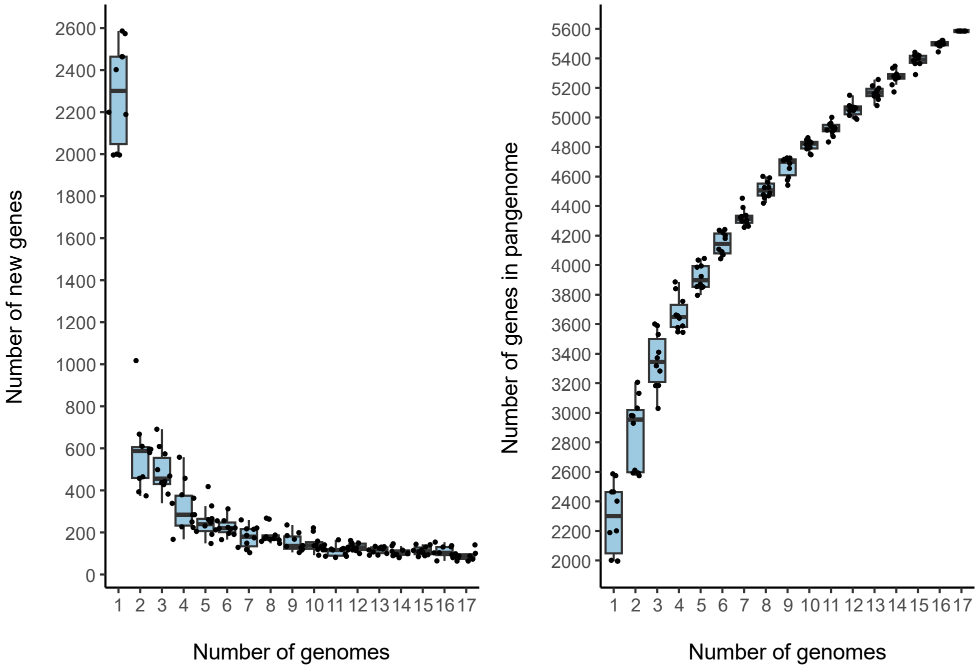

Supplementary Figure S9. Results of the pangenome analysis for each of the 30 species clusters presented in the manuscript. The pangenome of each species-cluster is shown in two diagrams. The plot on the left side shows the number of new genes that are added to the pangenome per genome included in the species cluster, while the plot on the right shows the succession of the total number of genes within the respective pangenome. Each data point represents one iteration of the MAFFT aligner used by the Roary software tool to create the core alignment between the genomes (MAGs) within the species clusters. The central line across the boxplots identifies the median, marking the dataset's midpoint. The box itself demarcates the interquartile range, extending from the first quartile to the third quartile, encapsulating the central 50% of the data. The whiskers project from the box to the furthest data points not categorized as outliers and show the spread of the main body of the dataset. Points located beyond the whiskers represent outliers, indicating data points that significantly deviate from the general distribution. *Illumatobacteraceae sp.1*1: n = 8, *Illumatobacteraceae sp.4*: n = 9, *Illumatobacteraceae sp.7*: n = 10, *Nanopelagicaceae sp.2*: n = 9, *Nanopelagicaceae sp.3*: n = 10, *Nanopelagicaceae sp.5*: n = 11, *Nanopelagicaceae sp.8*: n = 8, *Nanopelagicales sp.1*: n = 8, *Planktophila sp.10*: n = 8, *Planktophila sp.6*: n = 13, *Planktophila sp.9*: n = 10, *Bacteroidia sp.1*: n = 16, *Chitinophagaceae sp.2*: n = 12, *Chitinophagaceae sp.3*: n = 12, *Chitinophagaceae sp.4*: n = 10, *Chloroflexota sp.1*: n = 10, *Chloroflexota sp.2*: n = 13, *Chloroflexota sp.3*: n = 9, *Alphaprot. sp.1*: n = 10, *Burkholderiaceae sp.4*: n = 8, *Limnohabitans sp.6*: n = 9, *Polynucleobacter sp.3*: n = 18, *Polynucleobacter sp.5*: n = 8, *Rhodoferax sp.2*: n = 9, *Phycihaerales sp.1*: n = 16, *Lacunihaera sp.*3: n = 10, *Opitutaceae sp.2*: n = 11, *Opitutales sp.4*: n = 8, *Opitutales sp.5*: n = 11, *Opitutus sp.1*: n = 8. Raw data is provided as a Source Data file.
