# Supplementary material for: Freshwater genome-reduced bacteria exhibit pervasive episodes of adaptive stasis
Source: Nat Commun. 2024 Apr 23;15:3421. doi: 10.1038/s41467-024-47767-7 (PMC11039613; doi:10.1038/s41467-024-47767-7)
Supplement: Supplementary file 4 — Reporting Summary [file 41467_2024_47767_MOESM4_ESM.pdf]

nature portfolio

Corresponding author(s): Adrian-Stefan Andrei

Last updated by author(s): Apr 3, 2024

Reporting Summary

Nature Portfolio wishes to improve the reproducibility of the work that we publish. This form provides structure for consistency and transparency in reporting. For further information on Nature Portfolio policies, see our Editorial Policies and the Editorial Policy Checklist.

Statistics

For all statistical analyses, confirm that the following items are present in the figure legend, table legend, main text, or Methods section.

n/a	Confirmed	
☐	☒	The exact sample size (*n*) for each experimental group/condition, given as a discrete number and unit of measurement
☒	☐	A statement on whether measurements were taken from distinct samples or whether the same sample was measured repeatedly
☐	☒	The statistical test(s) used AND whether they are one- or two-sided<br>*Only common tests should be described solely by name; describe more complex techniques in the Methods section.*
☒	☐	A description of all covariates tested
☐	☒	A description of any assumptions or corrections, such as tests of normality and adjustment for multiple comparisons
☐	☒	A full description of the statistical parameters including central tendency (e.g. means) or other basic estimates (e.g. regression coefficient) AND variation (e.g. standard deviation) or associated estimates of uncertainty (e.g. confidence intervals)
☐	☒	For null hypothesis testing, the test statistic (e.g. *F*, *t*, *r*) with confidence intervals, effect sizes, degrees of freedom and *P* value noted<br>*Give P values as exact values whenever suitable.*
☒	☐	For Bayesian analysis, information on the choice of priors and Markov chain Monte Carlo settings
☒	☐	For hierarchical and complex designs, identification of the appropriate level for tests and full reporting of outcomes
☐	☒	Estimates of effect sizes (e.g. Cohen's *d*, Pearson's *r*), indicating how they were calculated

Our web collection on statistics for biologists contains articles on many of the points above.

Software and code

Policy information about availability of computer code

Data collection	No software was used for data collection.
Data analysis	Software:<br>BBMap v36.1<br>reformat.sh<br>bbduk.sh<br>bbmerge.sh<br>MEGAHIT v1.1.5<br>bbwrap.sh<br>jgi_summarize_bam_contig_depths<br>MetaBAT2<br>PRODIGAL v2.6.3<br>Mmseqs<br>CheckM v1.1.3<br>GTDB-Tk v1.4.0<br>HMMER hmmscan v3.1b2<br>pfam_scan.pl<br>InterProScan v5.24-63.0<br>Hmmsearch

BlastKOALA
MetQy
Prokka v.1.13
stripSubsetLCB
Blastn
Phobius v1.01
OrthoFinder v.2.5.2
bedtools v2.27.1
prank v.170427
IQ-TREE v 2.1.3
FEL v2.1
HYPHY 2.5.32
ColabFold v1.5.2
Chimera 1.17.1
R v4.0.3
RStudio v1.3.1093
GRiD v1.3
gRodon2
pipeline Roary v.3.13.0
mafft
inStrain
dRep
progressiveMauve v2.3.1
PhyML v3.3.3
ClonalFrameML v1.12

For manuscripts utilizing custom algorithms or software that are central to the research but not yet described in published literature, software must be made available to editors and reviewers. We strongly encourage code deposition in a community repository (e.g. GitHub). See the Nature Portfolio guidelines for submitting code & software for further information.

Data

Policy information about availability of data

All manuscripts must include a data availability statement. This statement should provide the following information, where applicable:

- Accession codes, unique identifiers, or web links for publicly available datasets
- A description of any restrictions on data availability
- For clinical datasets or third party data, please ensure that the statement adheres to our policy

All sequence data generated during this study have been deposited in the EBI/NCBI (Bioprojects: PRJEB35770, PRJEB35640, PRJNA428721, PRJNA429145). The accession numbers for the 52 raw metagenomic datasets are listed in Table S0 of the Supplementary Dataset. The 5 519 MAG IDs, their accession numbers, Bioproject IDs, and Sample IDs, along with additional metadata, are provided in Table S1 of the Supplementary Dataset. The generated data supporting the conclusions of this study may be found at figshare: 10.6084/m9.figshare.23546067. All additional important data supporting the study's conclusions are included in the publication and its supplemental material files. Source data are provided with this paper.
Public databases utilized in this study:
TIGRFAMs v15.0
Pfam release 32
NCBI NR
blastn
CDD v3.14
SMART v7.1
HAMAP v201701.18
COGs
KOfam
KEGG
SILVA87 138 SSU Ref NR 99
GTDB R05-RS95
UniProtKB/Swiss-Prot

Research involving human participants, their data, or biological material

Policy information about studies with human participants or human data. See also policy information about sex, gender (identity/presentation), and sexual orientation and race, ethnicity and racism.

Reporting on sex and gender	This study does not involve human research participants.
Reporting on race, ethnicity, or other socially relevant groupings	This study does not involve human research participants.
Population characteristics	This study does not involve human research participants.

Recruitment	This study does not involve human research participants.
Ethics oversight	This study does not involve human research participants.

Note that full information on the approval of the study protocol must also be provided in the manuscript.

Field-specific reporting

Please select the one below that is the best fit for your research. If you are not sure, read the appropriate sections before making your selection.

☐ Life sciences ☐ Behavioural & social sciences ☒ Ecological, evolutionary & environmental sciences

For a reference copy of the document with all sections, see nature.com/documents/nr-reporting-summary-flat.pdf

Ecological, evolutionary & environmental sciences study design

All studies must disclose on these points even when the disclosure is negative.

Study description	This is an exploratory metagenomic study focused on the recovery and analysis of environmental bacterial genomes. The nature of the study does not necessitate any treatment factors, interactions, design structure (factorial, nested, hierarchical) or replicates.
Research sample	Sample type: freshwater.<br>Sample number: 52.<br>Origin: Central European Lakes.<br>In response to the study's focus on microbial communities within freshwater ecosystems, our research sample comprises an extensive collection of datasets from metagenomic sequencing efforts targeting five distinct Central European lakes. These lakes were meticulously selected to represent a broad spectrum of trophic states, from oligotrophic (nutrient-poor) to dystrophic (rich in organic matter but low in oxygen), ensuring a comprehensive analysis of microbial diversity across different ecological conditions. This strategic selection was motivated by the hypothesis that varying nutrient availability and environmental pressures across these trophic states would influence the diversity and adaptive strategies of the resident bacterial populations.<br>The datasets themselves are a compilation of approximately 5,500 prokaryotic metagenome-assembled genomes (MAGs), generated from 52 shotgun-sequenced samples. This corresponds to about 11 billion reads and 3.31 Tb of data, making it one of the most substantial collections of lake microbial genomes analyzed to date.<br>Our rationale for this sample choice is twofold: to leverage the high-resolution insights provided by genome-resolved metagenomics for understanding bacterial adaptation and evolution, and to capture the diversity and dynamism of microbial communities across a gradient of environmental conditions. This approach allows us to dissect the complex interplay between genomic features, such as genome size, and ecological strategies within and among bacterial populations, shedding light on the underlying mechanisms of microbial diversification in freshwater habitats.
Sampling strategy	Samples from five freshwater lakes (that range in trophic status from oligotrophic to eutrophic; the Czech Republic and Switzerland) were used to recover genomic information from prokaryotes colonizing diverse freshwater niches.<br>Římov Reservoir (470m a.s.l., 48°50'N, 14°29'E, Czech Republic) is a meso-eutrophic, canyon-shaped dimictic water body with an area of 2.0km^2 (length 13.5km, the volume of 34.5×106 m^3, mean water retention time 77 days, maximum depth of 43m) that was built during 1974–1979 by damming a 13.5km long section of the River Malše. The sampling was performed between June 2015 and August 2017, above the deepest point of the reservoir by using a Friedinger sampler. 20L of water were collected from 0.5 (n=10) and 30m (n=8) depths and subjected to sequential peristaltic filtration through a series of 20, 5, and 0.2-µm-pore-size polycarbonate membrane filters (Sterlitech Corporation, USA). The sample collection and filtration steps were similar for the rest of the lakes/pools unless otherwise stated. Jiřická pond (892 m a.s.l., 48°36.96'N 14°40.59'E, Czech Republic) is a dystrophic humic water body with an area of 0.035 km2 (volume 6.59 x103 m^3, mean water retention time 9 days, maximum depth of 3.7 m), located in the Novohradské mountains of Southern Bohemia. Fifteen epilimnia (0.5 m depth) water samples were collected between May 2016 and August 2017. Lake Zurich (406m a.s.l., 47°18'N, 8°34'E, Switzerland) is an oligomesotrophic, perialpine monomictic water body, with an area of 67.3km^2 (length 40km, volume 3.3 km^3, mean water retention time 1.4 years, maximum depth of 136m). Thirteen samples were collected between 2013 – 2019 from the epilimnion (5 m depth, n=8) and hypolimnion (80/120 m depth, n=5) layers, and processed as described above. Lake Thun (558 m a.s.l., 46°41'N, 7°43'E, Switzerland) is an oligotrophic, alpine water body with an area of 48.3 km^2 (length 17.5 km, volume 6.5 km^3, mean water retention time 1.8 years, maximum depth of 217 m). Two water samples were collected in June 2018 from 5 and 180 m depths. Lake Constance (395 m a.s.l., 47°32'N, 9°31'E, Swiss Confederation) is an oligotrophic perialpine lake with an area of 473 km^2 (length 63 km, volume 48 km^3, mean water retention time 5 years, maximum depth of 252m). Four samples were collected in July and October 2018 from 5 m and 200 m depths.<br>The sampling locations within the lakes were selected based on their stratification (when applicable) to accurately reflect the varying environmental conditions. The volume of water collected aimed to encompass approximately 10^9 to 10^10 prokaryotic cells, with the specific quantity varying by lake, season, and individual sample. We did not perform any sample size calculations for this study.
Data collection	In our study, the data collection procedure was meticulously designed to capture a comprehensive snapshot of prokaryotic diversity across different freshwater ecosystems.<br>1. Selection of Sampling Sites: We strategically chose sampling locations within each of the five Central European lakes to reflect the unique ecological stratification present within these bodies of water. This approach ensured that the collected samples represented the broad range of microenvironments and the microbial life they harbor.<br>2. Seasonal and Spatial Sampling: Recognizing the influence of seasonal changes on microbial communities, we conducted sampling across various seasons. Additionally, we selected multiple sites within each lake to account for spatial heterogeneity, aiming to encompass the full spectrum of microbial diversity.

3. Volume of Water Collected: The volume of water sampled at each site was carefully determined based on the expected prokaryotic cell density, which varies significantly depending on factors like the lake's trophic status, the season, and specific lake conditions at the time of sampling. Our target was to collect water volumes containing approximately 10^9 to 10^10 prokaryotic cells, optimizing the yield of metagenomic DNA for sequencing.

4. Metagenomic Sequencing: From the collected water samples, we extracted metagenomic DNA, which was then subjected to high-throughput shotgun sequencing. This process allowed us to generate vast datasets, capturing the genetic material of the prokaryotic communities present.

By adhering to this detailed procedure, we aimed to ensure the reliability and representativeness of our data, providing a solid foundation for our analyses of microbial diversity and evolutionary dynamics in freshwater lakes.

Persons involved in data collection: Vinicius Kavagutti, Maliheh Mehrshad, Tanja Shabarova, Michaela Salcher, Petr Znachor, Pavel Rychteck, Petr Porcal, Thomas Posch, Eugen Loher and Adrian-Stefan Andrei.

Several multi-parametric probes were deployed to profile the physicochemical characteristics of the sampled lakes. DNA was extracted from the 0.22-µm filters (0.2- to 5-µm fraction) using the ZR Soil Microbe DNA MiniPrep kit (Zymo Research, Irvine, CA, USA) in accordance with the manufacturer's instructions. The total quantity of DNA was estimated using the Qubit dsDNA BR assay kit (Life Technologies, Foster City, CA, USA) on a Qubit 2.0 fluorometer (Life Technologies). DNA integrity was assessed by agarose gel (2%) electrophoresis and SYBR green I stain. Shotgun sequencing was performed using the Novaseq 6000 sequencing platform (2× 150 bp) (Novogene, Hong Kong, China).

Timing and spatial scale

Sample Name Date Depth (m) Location
ZH-Oct18 Oct.18 120 Lake Zurich
ZH-Jul18 Jul.18 120 Lake Zurich
ZH-3nov15 Nov.15 80 Lake Zurich
ZH-15may19 May.19 120 Lake Zurich
ZH-13may13 May.13 80 Lake Zurich
ZE05-15may19 May.19 5 Lake Zurich
ZE-Oct18 Oct.18 5 Lake Zurich
ZE-Jul18 Jul.18 5 Lake Zurich
ZE-3nov15 Nov.15 5 Lake Zurich
ZE-17mar17 Mar.17 5 Lake Zurich
ZE-15may19 May.19 5 Lake Zurich
ZE-13oct10 Oct.10 5 Lake Zurich
ZE-13may13 May.13 5 Lake Zurich
TH-Jun18 Jun.18 180 Lake Thun
TE-Jun18 Jun.18 5 Lake Thun
RH-9nov16 Nov.16 30 Rimov Reservoir
RH-26jul17 Jul.17 30 Rimov Reservoir
RH-23may17 May.17 30 Rimov Reservoir
RH-20apr16 Apr.16 30 Rimov Reservoir
RH-18aug17 Aug.17 30 Rimov Reservoir
RH-15aug16 Aug.16 30 Rimov Reservoir
RH-27jun17 Jun.17 30 Rimov Reservoir
RH-14apr17 Apr.17 30 Rimov Reservoir
RE-9nov16 Nov.16 0.5 Rimov Reservoir
RE-4nov15 Nov.15 0.5 Rimov Reservoir
RE-27jun17 Jun.17 0.5 Rimov Reservoir
RE-26jul17 Jul.17 0.5 Rimov Reservoir
RE-23may17 May.17 0.5 Rimov Reservoir
RE-20apr16 Apr.16 0.5 Rimov Reservoir
RE-18aug17 Aug.17 0.5 Rimov Reservoir
RE-16jun15 Jun.15 0.5 Rimov Reservoir
RE-15aug16 Aug.16 0.5 Rimov Reservoir
RE-14apr17 Apr.17 0.5 Rimov Reservoir
Jr-7aug17 Aug.17 0.5 Jiřická pond
Jr-29jan18 Jan.18 0.5 Jiřická pond
Jr-28jun17 Jun.17 0.5 Jiřická pond
Jr-28aug17 Aug.17 0.5 Jiřická pond
Jr-25may17 May.17 0.5 Jiřická pond
Jr-23apr18 Apr.18 0.5 Jiřická pond
Jr-20nov17 Nov.17 0.5 Jiřická pond
Jr-19feb18 Feb.18 0.5 Jiřická pond
Jr-16oct17 Oct.17 0.5 Jiřická pond
Jr-14may18 May.18 0.5 Jiřická pond
Jr-12mar18 Mar.18 0.5 Jiřická pond
Jr-11may16 May.16 0.5 Jiřická pond
Jr-11dec17 Dec.17 0.5 Jiřická pond
Jr-08jan18 Jan.18 0.5 Jiřická pond
Jr-03apr18 Apr.18 0.5 Jiřická pond
CH-oct18 Nov.18 200 Lake Constance
CH-jul18 Jul.18 200 Lake Constance
CE-oct18 Oct.18 5 Lake Constance

	CE-jul18 Jul.18 5 Lake Constance
Data exclusions	No data were excluded from the analyses.
Reproducibility	The data generated are available in referenced public repositories, ensuring transparency and accessibility. All methods used in this study are extensively cited, and the parameters for software applications are fully documented. Given the study's reliance on environmental samples, no attempts were made to replicate the experiments.
Randomization	This is an exploratory study and randomization is not relevant to the study design.
Blinding	Blinding was not performed because it was not relevant to this study. This study was an exploratory survey of microbial diversity without a priori expectations that would influence the analyses.

Did the study involve field work? [x] Yes [ ] No

Field work, collection and transport

Field conditions	Water temperatures varied from 0.2°C to 24.1°C, influenced by the specific lake and season. For comprehensive temperature data, refer to Table S1 in the Supplementary Dataset. Weather conditions at the time of sampling were typical for each location, and no sampling occurred during weather anomalies.
Location	Římov Reservoir (470m a.s.l., 48°50′N, 14°29′E, Czech Republic).<br>Jiřická pond (892 m a.s.l., 48.616034 N 14.676594 E, Czech Republic).<br>Lake Zurich (406m a.s.l., 47°18′N, 8°34′E, Switzerland).<br>Lake Thun (558 m a.s.l., 46°41'N, 7°43'E, Switzerland).<br>Lake Constance (395 m a.s.l., 47°32'N, 9°31'E, Switzerland).
Access & import/export	All samples were collected in compliance with local, cantonal and national laws. No permits were required for lake sampling. Sampling at Lake Zurich was conducted in collaboration with the University of Zurich's Limnological Station. Lake Thun sampling occurred in partnership with the Canton of Bern's Laboratory for Water and Soil Protection. Collaboration for Lake Constance involved the Institute for Lake Research (ISF) of the State Agency for Environment Baden-Württemberg, Germany. The Jiřická Pond and Rimov Reservoir sampling received support from the Institute of Hydrobiology of the Czech Academy of Sciences. Data processing for Lakes Zurich, Thun, and Constance occurred within Switzerland, while processing for Jiřická Pond and Rimov Reservoir was in the Czech Republic, eliminating the need for export/import permits. The last author was previously affiliated with the Institute of Hydrobiology of the Czech Academy of Sciences and is currently with the University of Zurich's Limnological Station.
Disturbance	No disturbances were caused by the sampling procedures.

Reporting for specific materials, systems and methods

We require information from authors about some types of materials, experimental systems and methods used in many studies. Here, indicate whether each material, system or method listed is relevant to your study. If you are not sure if a list item applies to your research, read the appropriate section before selecting a response.

Materials & experimental systems

n/a	Involved in the study	
[x]	[ ]	Antibodies
[x]	[ ]	Eukaryotic cell lines
[x]	[ ]	Palaeontology and archaeology
[x]	[ ]	Animals and other organisms
[x]	[ ]	Clinical data
[x]	[ ]	Dual use research of concern
[x]	[ ]	Plants

Methods

n/a	Involved in the study	
[x]	[ ]	ChIP-seq
[x]	[ ]	Flow cytometry
[x]	[ ]	MRI-based neuroimaging
